# Genome‐enabled discovery of candidate virulence loci in *Striga hermonthica*, a devastating parasite of African cereal crops

**DOI:** 10.1111/nph.18305

**Published:** 2022-07-07

**Authors:** Suo Qiu, James M. Bradley, Peijun Zhang, Roy Chaudhuri, Mark Blaxter, Roger K. Butlin, Julie D. Scholes

**Affiliations:** ^1^ School of Biosciences University of Sheffield Western Bank Sheffield S10 2TN UK; ^2^ Institute of Evolutionary Biology, School of Biological Sciences The University of Edinburgh, Ashworth Laboratories Charlotte Auerbach Road Edinburgh EH9 3FL UK; ^3^ Wellcome Sanger Institute Wellcome Genome Campus, Hinxton Cambridge CB10 1SA UK; ^4^ Department of Marine Sciences University of Gothenburg S‐405 30 Gothenburg Sweden

**Keywords:** parasitic plants, population genomics, secretome, *Striga* genome, *Striga hermonthica*, virulence factors (VFs)

## Abstract

- Parasites have evolved proteins, virulence factors (VFs), that facilitate plant colonisation, however VFs mediating parasitic plant–host interactions are poorly understood. *Striga hermonthica* is an obligate, root‐parasitic plant of cereal hosts in sub‐Saharan Africa, causing devastating yield losses. Understanding the molecular nature and allelic variation of VFs in *S. hermonthica* is essential for breeding resistance and delaying the evolution of parasite virulence.
- We assembled the *S. hermonthica* genome and identified secreted proteins using *in silico* prediction. Pooled sequencing of parasites growing on a susceptible and a strongly resistant rice host allowed us to scan for loci where selection imposed by the resistant host had elevated the frequency of alleles contributing to successful colonisation.
- Thirty‐eight putatively secreted VFs had very different allele frequencies with functions including host cell wall modification, protease or protease inhibitor and kinase activities. These candidate loci had significantly higher Tajima's *D* than the genomic background, consistent with balancing selection.
- Our results reveal diverse strategies used by *S. hermonthica* to overcome different layers of host resistance. Understanding the maintenance of variation at virulence loci by balancing selection will be critical to managing the evolution of virulence as part of a sustainable control strategy.

## Abstract 2

See also the Commentary on this article by Westwood, **236**: 316–318.

## Introduction

Plants are constantly challenged by diverse parasites. As a consequence, they have evolved sophisticated surveillance systems to detect and protect themselves against parasite invasion (Wu *et al*., 2018; Kanyuka & Rudd, 2019). In turn, plant parasites have evolved suites of proteins, miRNAs, or other molecules that are delivered into host plants to facilitate colonisation (virulence factors (VFs)) (Win *et al*., 2012; Mitsumasu *et al*., 2015; Ceulemans *et al*., 2021; Mitchum & Liu, 2022) and they are pivotal in determining the outcome of a parasite–plant interaction.

Parasitic plants have evolved independently at least 12 times (Kuijt, 1969; Westwood *et al*., 2010). Regardless of evolutionary origin, parasitic plants possess a multicellular organ called the ‘haustorium’, through which direct structural and physiological connections are formed with their host plant (Westwood, 2013; Yoshida *et al*., 2016). This allows them to abstract water, organic and inorganic nutrients. In addition, the haustorium is increasingly recognised to play a role in host manipulation, through the movement of parasite VFs into the host plant (Shahid *et al*., 2018; Clarke *et al*., 2019). An example is provided by a particular ‘race’ of *Striga gesnerioides*, which delivers a small, secreted leucine‐rich repeat (LRR) domain‐containing effector (Suppressor of Host Resistance 4z (SHR4z)) into cowpea host cells, whereupon it triggers rapid turnover of the E3 ubiquitin ligase, VuPOB1, a positive regulator of the host's defence response (Su *et al*., 2020).

*Striga* is a genus of obligate, root‐parasitic plants within the Orobanchaceae (Parker & Riches, 1993; Spallek *et al*., 2013). One species in particular, *Striga hermonthica*, infests rain‐fed rice, maize, sorghum and millets, leading to devastating losses in crop yields for resource‐poor farmers in sub‐Saharan Africa (Scholes & Press, 2008; Rodenburg *et al*., 2016). Control of *S. hermonthica* is extremely difficult as the parasite is an obligate outbreeder, with high fecundity, wide dispersal and a persistent, long‐lived seed bank (Parker & Riches, 1993) leading to a large effective population size (Huang *et al*., 2012). Resistant crop varieties are a crucial component of successful control strategies (Scholes & Press, 2008) however, even for crop varieties considered highly resistant, genetic variation within parasite populations is such that a few individuals can overcome host resistance and form successful attachments (Gurney *et al*., 2006; Cissoko *et al*., 2011). To develop crop varieties with durable resistance against *S. hermonthica*, it is vital to understand the repertoire, mode of action and genetic variability of parasite VFs (Timko *et al*., 2012; Rodenburg *et al*., 2017). Given the highly polymorphic populations of *S. hermonthica* and genetic diversity of the seed bank, we hypothesised that *S. hermonthica* is likely to possess suites of VFs that allow it to overcome layers of resistance in multiple host plant varieties. The aim of this study was to discover candidate genes encoding polymorphic VFs in *S. hermonthica*.

**Fig. 1:**
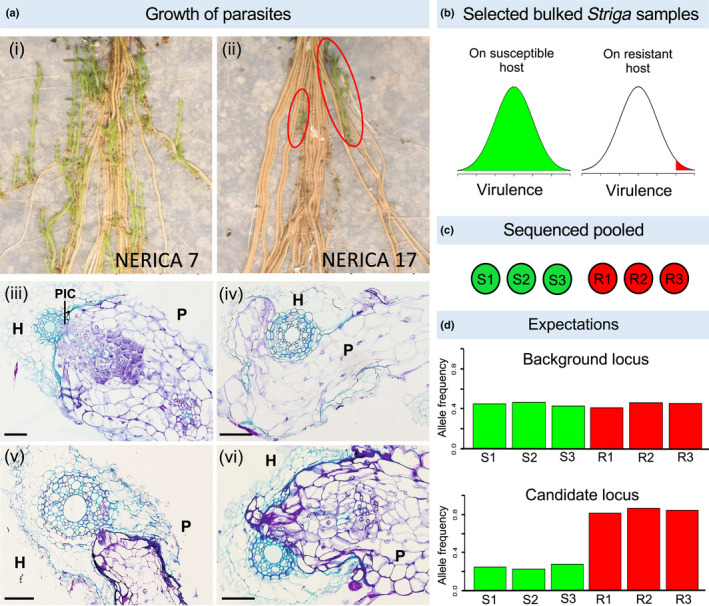
Experimental strategy for the identification of the *Striga hermonthica* virulence loci. *Striga hermonthica* (Kibos accession) were grown on susceptible (NERICA 7) and resistant (NERICA 17) rice hosts (a). The whole rice root systems show many *S. hermonthica* individuals parasitising the roots of NERICA 7 (i) whilst only two individuals (red circles) were able to overcome the resistance response of NERICA 17 (ii). Transverse sections show *S. hermonthica* invading rice roots for a representative susceptible (iii) and resistant (iv–vi) interaction 7 d after inoculation. In the successful host–parasite interaction parasite intrusive cells (PIC) have breached the endodermis and have made connections with the host's xylem (iii). In the resistant rice variety several phenotypes are observed; The parasite invades the host root cortex but is unable to penetrate the suberised endodermis (iv, v); the parasite penetrates the endodermis but is unable to form connections with the host xylem (vi). H, host root; P, parasite. Bars, 5 μm. Our experimental strategy was based on the prediction that many *S. hermonthica* genotypes would grow on NERICA 7 but only highly virulent genotypes would grow on NERICA 17 (b). Samples of 100 *S. hermonthica* plants were bulked to generate three sequencing pools from each host variety (c). We expected that background loci would not differ in allele frequency between pools, but virulence alleles (and neutral alleles in linkage disequilibrium) would have increased frequency in all pools from the resistant host, allowing us to identify candidate loci (d). S1–S3, sequencing pools from susceptible plants (NERICA‐7); R1–R3, sequencing pools from resistant plants (NERICA‐17).

To achieve our aims we combined two complementary approaches. First, we assembled and annotated the genome of *S. hermonthica*, and developed a pipeline for computational prediction of putative secreted proteins (the secretome) and candidate VFs. The assembled genome was then used as a reference for an experimental, population genomics analysis, to compare DNA sequence variants in bulked (pooled) samples of *S. hermonthica* grown on a susceptible (NERICA‐7) or resistant (NERICA‐17) rice host (Fig. 1a i,ii). This allowed us to scan for loci in the *S. hermonthica* genome where the selection imposed by the resistant host had elevated the frequency of alleles contributing to successful colonisation (termed ‘virulence’ alleles) (Fig. 1b–d). A similar approach was used to identify candidate genomic regions associated with resistance in *Solanum vernei* to the potato cyst nematode, *Globodera pallida* (Eoche‐Bosy *et al*., 2017). The intersection between genes encoding predicted VFs and genes with highly significant allele frequency differences in the genome scan of *S. hermonthica*, revealed a set of candidate virulence loci encoding proteins with many functions, for example, cell wall modification, protease or protease inhibitor and receptor‐like protein kinase activities. Our results suggest that diverse strategies are used by *S. hermonthica* to overcome different layers of host resistance, resulting in a polygenic basis of virulence in this parasite.

## Materials and Methods

### Collection and extraction of *S. hermonthica* DNA

An accession (population sample) of *S. hermonthica* (Del.) Benth. seeds was collected from individuals parasitising maize in farmers' fields in the Kibos region of Kenya (0°5′30″S, 34°46′4″E). To obtain *S. hermonthica* for genome sequencing and the bulked sample analysis (BSA), rice seedlings of the varieties, NERICA‐7 and NERICA‐17, were grown in rhizotrons and infected with germinated *S. hermonthica* seeds (Gurney *et al*., 2006). Plants were grown in a 12 h photoperiod, a photon‐flux density of 500 μmol quanta m^−2^ s^−1^ at plant height, a day : night temperature of 28°C : 25°C and 60% relative humidity. For the construction of a reference genome, one *S. hermonthica* individual was randomly harvested from NERICA‐7. For the pooled sequencing, 300 *S. hermonthica* individuals (> 30 mg in weight) were harvested from NERICA‐7 and NERICA‐17, divided into 20 mg aliquots and immediately frozen in liquid nitrogen. The 300 individuals from NERICA‐7 and NERICA‐17 were divided into three pools of 100 individuals (biological replicates). DNA was extracted from the six pools (Supporting Information Methods S1) and samples were subjected to paired‐end sequencing using Illumina HiSeq at the Beijing Genomics Institute, China. The libraries, insert sizes and sequencing depth are shown in Table S1. DNA from the individual harvested from NERICA‐7, for the production of a reference genome, was sequenced on an Illumina HiSeq 2500 system at Edinburgh Genomics, UK. Six paired‐end DNA libraries were constructed with different insert sizes (Table S1).

### *De novo* assembly of the *S. hermonthica* genome

Reads were cleaned and filtered (Methods S1). After filtering, *c*. 2.7 billion reads were generated from the short insert libraries and 0.76 billion reads from mate‐pair libraries. This corresponded to *c*. 230× and *c*. 54× coverage of the *S. hermonthica* genome, respectively. The cleaned and filtered reads were used to assess the *S. hermonthica* genome size, repetitiveness and heterozygosity, compared with 12 other plant species (Table S2), in the module preQC, implemented in the software Sga (https://github.com/jts/sga). This analysis showed *S. hermonthica* was highly heterozygous and therefore the software Platanus, which is specifically designed for highly heterozygous genomes, was chosen to assemble the *S. hermonthica* genome (Kajitani *et al*., 2014) (Table S3).

To further improve the *S. hermonthica* genome assembly, Chicago and Dovetail Hi‐C libraries were prepared and sequenced at Dovetail Genomics, CA, USA (https://dovetailgenomics.com/plant‐animal/) (Table S3). For the Chicago libraries, DNA from the *S. hermonthica* individual used for the genome, was sequenced on an Illumina HiSeq 2500 system. For the Hi‐C libraries, an F1 individual from a cross between the genome individual and another Kibos individual was used. The Chicago and Hi‐C libraries were used only to improve the contiguity of the initial genome assembly, using the Dovetail HiRise Assembler software. RepeatModeler was used to generate a *S. hermonthica*‐specific repeat library and RepeatMasker was then used to classify repeat elements in the genome. A repeat‐masked version of the genome was used for annotation (Smit & Hubley, 2008; Smit *et al*., 2013).

### Annotation of the *S. hermonthica* genome

The genome was annotated using three methods (full details in Methods S1). First, gene structures were inferred using a *S. hermonthica* transcriptome dataset of cDNAs collected from *S. hermonthica* individuals at eight developmental stages, generated by the Parasitic Plant Genome Project (PPGP) (Westwood *et al*., 2012). Second, protein sequences from *Arabidopsis thaliana* (TAIR10), *Mimulus guttatus* (v.2.0), *Solanum lycopersicum* (ITAG2.4), *Oryza sativa* (IRGSP1.0) and *Sorghum bicolor* (79), were used to determine consensus gene models in the genome. Third, an *ab initio* method was used for *de novo* prediction of genes in the *S. hermonthica* genome using the software, Braker, with default settings (Hoff *et al*., 2016). Finally, Evidence Gene Modeler was used to integrate various gene models from these approaches (Haas *et al*., 2008). The completeness of the gene set was assessed using Busco v.5 using the 2326 core orthologues from eudicots_odb10, with default settings. Missing Busco IDs for *Striga* and *Cuscuta* genomes were queried against OrthoDB v.10.1 (Kriventseva *et al*., 2019) to retrieve the corresponding Gene Ontology (GO) terms. Enrichment of GO terms was tested using a chi‐squared test against a background of GO terms obtained for the complete set of Buscos in eudicots_odb10, with the *chisq.test* function in R (R Core Team, 2021).

Putative protein functions were assigned to *S. hermonthica* proteins using Blastp analyses against the SwissProt and TrEMBL databases, and against the proteomes of *A. thaliana* (v.30) and *O. sativa* (v.7). A Blastp analysis was also conducted against the pathogen–host interaction database (PHI‐base, v.4.2) (http://www.phi‐base.org/index.jsp). Blastp analyses were run locally using the NCBI Blast package (version: ncbi‐2.3.0+) and a hit was taken to be significant if *e*‐value < 10^−5^, bit score and percentage identity > 30. Protein motifs and domains were determined by searching databases including Pfam, PANTHER, GENE3D, CDD, PRINTS, PROSITE, ProDom and SMART with InterProScan GO terms for individual proteins retrieved from the corresponding InterPro descriptions.

Orthologous gene groups (OGs) were inferred using the software OrthoFinder v.2 (Emms & Kelly, 2015). The number of genes per species for each OG was transformed into a matrix of *Z*‐scores to quantify gene family expansion/contraction. The significance of expansion or contraction was determined using Café v.4.2 (Han *et al*., 2013). Functional annotation of OGs was predicted based on sequence similarity to the InterPro protein family database (please refer to Methods S1).

### Prediction, analysis and refinement of the *S. hermonthica* secretome

Secreted *S. hermonthica* proteins were predicted using SignalP v.3.0 and 4.1 (Bendtsen *et al*., 2004; Petersen *et al*., 2011) (Fig. S1). Transmembrane spanning regions were identified using Tmhmm2.0 (Krogh *et al*., 2001). Proteins with a secretion signal but without a predicted transmembrane helix were retained as the ‘secretome’. Pfam domains enriched in the *S. hermonthica* secretome compared with the rest of the proteome (nonsecretome) were significant when the corrected *P‐*value was < 0.1, according to a chi‐squared test with a false discovery rate (FDR) correction for multiple testing (Benjamini & Hochberg, 1995). The initial secretome was then refined into subsets based on a series of structural and functional characteristics (Fig. S1) (details in Methods S1).

### Identification and analysis of candidate virulence loci using pooled sequencing data

The raw sequence reads from the six pools were trimmed and filtered for coverage (please refer to Methods S1). The likelihood of the observed read counts for the two most common alleles, across the six pools was calculated according to eqn 3 from Gompert & Buerkle (2011) to allow for the two levels of sampling associated with pooled sequencing data (sampling of reads and of individuals). We compared three allele frequency models for each single nucleotide polymorphism (SNP) using the Akaike information criterion (AIC): a null model with a single allele frequency for all pools, a control‐virulent model with one frequency for the control pools (from the NERICA‐7 host) and one for the virulent pools (from the NERICA‐17 host) and a replicate model with a different allele frequency for each of the three pairs of pools (one control and one virulent) that were sequenced together. The control‐virulent model was the model of interest whilst the replicate model was intended to check for consistency across pairs of pools. Therefore, two ΔAIC values were obtained: ΔAICcv = AICnull − AICcontrol‐virulent and ΔAICrep = AICcontrol‐virulent − AICreplicate. High positive values of ΔAICcv represent better fits compared with the null model and indicate significant differences between control and virulent pool types. SNPs with positive ΔAICrep values were likely to be affected by artefacts caused by sequencing methods and were excluded from the following analyses. All analysis steps were repeated independently for SNPs based on Bwa and Novoalign mapping as recommended by Kofler *et al*. (2016).

The effective population size in *Striga* is likely to be large (Parker & Riches, 1993) and this is consistent with high diversity in our samples (overall mean π = 0.011). Therefore, we also expected that linkage disequilibrium would break down quickly. To define a suitable window size to search for regions potentially implicated in virulence, the extent of linkage disequilibrium in *S. hermonthica* was investigated (please refer to Methods S1 for details). On the basis of this analysis (Fig. S2), 1 kbp windows were used to detect genomic regions potentially associated with virulence on the basis of allele frequency differences between pools from the susceptible and resistant hosts. Permutation tests were then used to detect candidate genes with outlying levels divergence (described in Methods S1).

Two population statistics were calculated for each genic region in the control pool using the software Popoolation (Kofler *et al*., 2011) (details in Methods S1). These were nucleotide diversity (π) and Tajima's *D*, a statistic describing the allele frequency spectrum used for testing whether a DNA sequence is evolving under a process that departs from the standard neutral model, such as selection or demographic change (Tajima, 1989).

The candidate virulence genes were categorised into functional groups based on the annotations of the closest matching homologs from the *A. thaliana* and *O. sativa* proteomes, as well as the Pfam domain annotations. For each gene, the numbers of SNPs were counted for the promoter region (within 2 kbp upstream of the start codon), the intronic region and coding region, and the numbers of nonsynonymous SNPs were determined. To quantify the allele frequency differences between control and virulent pools for these candidate virulence genes, the proportion of SNPs with high fixation index (*F*_ST_) values in the significant window was calculated (please refer to Methods S1).

### Expression profiling of candidate virulence genes

Expression profiles for candidate virulence genes were determined for *S. hermonthica* collected at 2, 4, or 7 d post inoculation (dpi) from the roots of NERICA‐7 rice plants (full details in Methods S1). In addition, unattached *S. hermonthica* haustoria were induced *in vitro* by the addition of 10 μM DMBQ (Fernández‐Aparicio *et al*., 2013). Cleaned reads were mapped to the *S. hermonthica* genome using Tophat2 v.2.0.12 and quantified with HTSeq (v.0.6.1). FPKM values for each gene at each time point were used to calculate a fold change in expression relative to the haustorial sample and significance assessed with a one‐way ANOVA using the *aov* function in R (R Core Team, 2021). For each gene, log_2_ fold expression values, across the time points, were centred around 0 and scaled by the standard deviation for plotting as a heatmap using the *pheatmap* function in R. Further details are provided in Methods S1.

## Results

### The *S. hermonthica* genome is very heterozygous

**Fig. 2:**
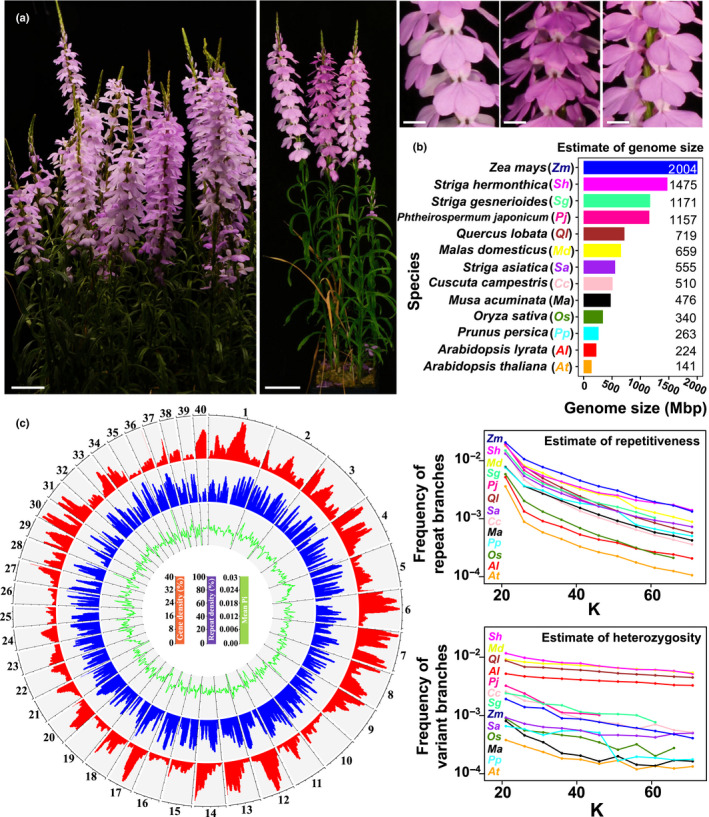
*Striga hermonthica* is an obligate outbreeding parasitic plant with a highly heterozygous and repetitive genome. (a) Flowering *S. hermonthica* growing on the rice host, NERICA‐7, derived from a seed batch collected from the Kibos region of Kenya. Middle image shows three flowering *S. hermonthica* individuals parasitising the same rice host plant (the browned leaves of rice plant are visible at the base) (bars, 5 cm). Images to the right are magnified versions of individual flowers from parasites shown in the middle image (bars, 1 cm). (b) Comparison of genome size, heterozygosity and repetitiveness between *S. hermonthica* and 12 other plants (Supporting Information Table S2). The estimate of the genome size (Mbp) was based on k‐mer count statistics. The estimate of heterozygosity was based on variant branches in the k‐de Bruijn graph. The repetitiveness of the genomes was based on frequency of repeat branches in the k‐de Bruijn graph. K, k‐mer length. (c) Genomic features calculated in 1 Mbp windows with a slide of 250 kbp for the largest 40 scaffolds in the *S. hermonthica* genome assembly. Outer bar plot (red): gene density (percentage of the window comprised of genic regions). Mid bar plot (blue): repeat density (percentage of window comprised of repetitive sequence). Inner line plot (green): nucleotide diversity (mean Pi for genic regions). Axes tick marks around plot circumference denote 4 Mbp. Vertical axis tick marks are defined in the centre.

We obtained a single population of *S. hermonthica* seeds from farmer's fields in Kibos, Kenya and infected a highly susceptible rice variety, NERICA‐7 (Fig. 1a). The genetic diversity of the seed population is reflected in the subtle differences of flower colour of attached parasites (Fig. 2a). We sequenced, assembled and characterised the genome of a single individual from this population. The genome size was estimated by k‐mer analysis to be 1475 Mbp (Fig. 2b), in close agreement with a flow cytometry‐based estimate (Estep *et al*., 2012) and more than twice the size of the genome of *S. asiatica* (Yoshida *et al*., 2019). The assembly consisted of 34 907 scaffolds > 1 kbp in length, with an N50 of 10.0 Mbp and 29 scaffolds making up half of the genome size (Table S3). The *S. hermonthica* genome was markedly heterozygous (overall mean π = 0.011) (Fig. 2c) when compared with other parasitic and nonparasitic plant genomes, likely to reflect the fact that it is an obligate outbreeding species. In addition, the genome contained a large proportion (69%) of repetitive DNA (Fig. 2b,c), dominated by long terminal repeat (LTR) elements (Table S4), a pattern also found for the shoot‐parasitic plants, *Cuscuta australis* and *C. campestris* (Sun *et al*., 2018; Vogel *et al*., 2018) and the closely related parasitic plant *S. asiatica* (Yoshida *et al*., 2019). As expected, the density of repetitive elements along each scaffold negatively correlated with the density of protein‐coding genes (Fig. 2c). In total, 29 518 protein‐coding genes were predicted from the *S. hermonthica* genome, which was comparable with *S. asiatica* (34 577), the closely related nonparasitic plant *M. guttatus* (28 140) and to *A. thaliana* (27 416) (Table S5).

**Fig. 3:**
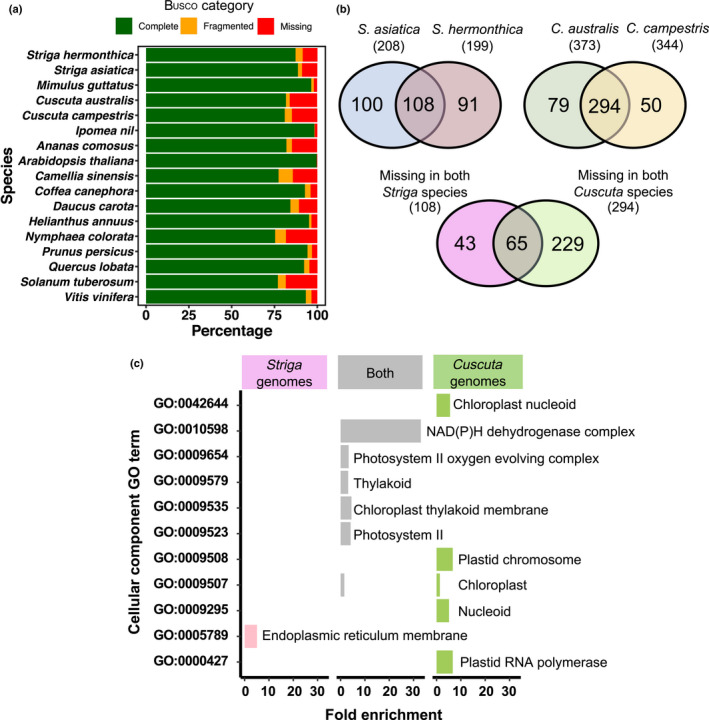
(a) Busco completeness analysis for the *Striga hermonthica* genome, compared with 16 other published plant genomes. (b) The number of missing Buscos for two *Striga* and two *Cuscuta* species. The overlaps show the number of missing Buscos from both *Striga* or both *Cuscuta* species, respectively. The overlap between these sets shows the number of missing Buscos from all four parasitic plant genomes. (c) Significantly enriched (chi‐squared test, false discovery rate (FDR) corrected *P*‐value < 0.05) GO terms for the namespace ‘cellular component’ associated with Buscos missing in only the two *Striga* genomes (pink), only the two *Cuscuta* genomes (green) or both *Striga* and both *Cuscuta* genomes (grey).

Busco analysis of gene set completeness (Waterhouse *et al*., 2018), showed 87.3% of 2326 conserved single‐copy orthologues in eudiocotyledons were complete in the *S. hermonthica* genome, similar to that found in *S. asiatica* (88.7%) (Fig. 3a; Table S6). Of the Buscos not found in the *S. hermonthica* genome, over half were also absent from the *S. asiatica* genome (Fig. 3b; Table S6). Both *Striga* spp. shared missing Buscos that were present in the genome of the closely related nonparasitic *M. guttatus* (Fig. 3b; Table S6). Similarly, two shoot holoparasites, *C. australis* and *C. campestris*, with a Busco completeness of 81.0% and 81.7% respectively, also shared many missing Buscos that were present in the genome of their nonparasitic relative, *Ipomea nil* (Fig. 3b). Of the Buscos missing from the *Striga* or *Cuscuta* genomes, 65 were missing from all four parasitic plants (Fig. 3b) and these were enriched with GO terms related to the chloroplast and photosynthesis (Fig. 3c; Table S7). This is consistent with previous findings suggesting some missing Buscos are likely to be a result of the parasitic lifestyle (Sun *et al*., 2018; Vogel *et al*., 2018; Yoshida *et al*., 2019; Cai *et al*., 2021).

**Fig. 4:**
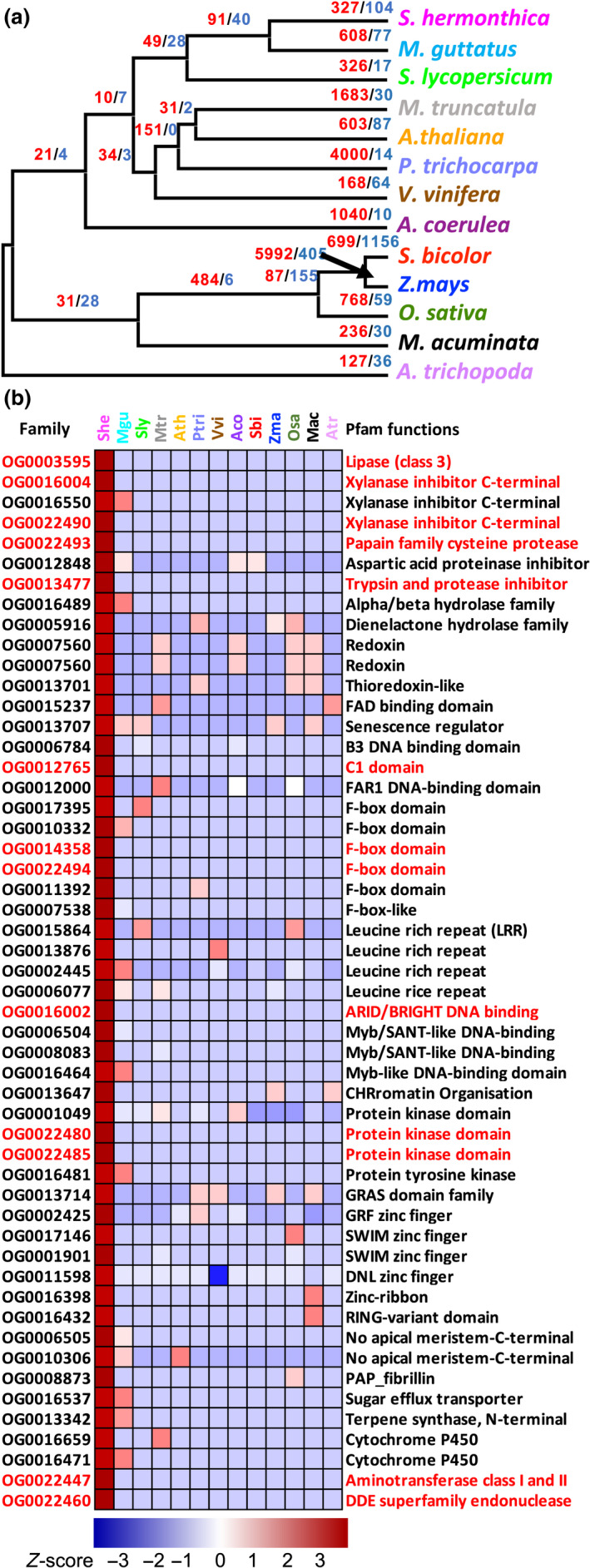
Orthogroup analyses. (a) An ultrametric tree for *Striga hermonthica* and 12 other species generated in Mega, based on 42 single‐copy genes inferred from OrthoFinder. The original maximum likelihood tree with branch support is shown in Supporting Information Fig. S3. The number of significantly expanded (red) and contracted (blue) orthogroups based on Café analysis are shown above the branches. (b) Significantly expanded orthogroups in *S. hermonthica*, after removing proteins encoded as transposable elements, compared with 12 other plant species. Orthogroups only found in *S. hermonthica*, have family names in red. Higher *Z*‐scores indicate the orthogroups are more expanded in a species whilst lower *Z*‐scores indicate the orthogroups are more contracted in a species.

Comparative analysis of OGs (orthogroups) between *S. hermonthica* and 12 other plant species identified 22 624 orthogroups in total, of which 12 278 contained *S. hermonthica* genes. Of these, 327 were significantly expanded and 104 were contracted in the *S. hermonthica* genome, identified by Café analyses (Fig. 4a). Low branch supports due to short branch lengths were observed for the clade consisting of *P. trichocarpa*, *V. vinifera*, *M. truncatula* and *A. thaliana* (Fig. S3). This might influence the numbers of significantly expanded and contracted orthogroups for these four species but it is less likely to affect the results in *S. hermonthica*, especially the most expanded orthogroups shown in Fig. 4(b). Expanded orthogroups included the α/β‐hydrolase family, recently shown to have undergone duplication in *S. hermonthica* (Toh *et al*., 2015), as well as numerous F‐box, LRR and protein kinase domain‐containing proteins (Fig. 4b). Of particular interest in the context of pathogenicity were *S. hermonthica*‐specific orthogroups annotated as papain family cysteine proteases, xylanase inhibitors and trypsin and protease inhibitors (Fig. 4b). Both proteases and protease inhibitors function in a wide range of plant–plant parasite interactions and may act offensively, by degrading host proteins or defensively, by inhibiting host defence enzymes (Bleischwitz *et al*., 2010; Mueller *et al*., 2013).

### The *S. hermonthica* secretome

**Fig. 5:**
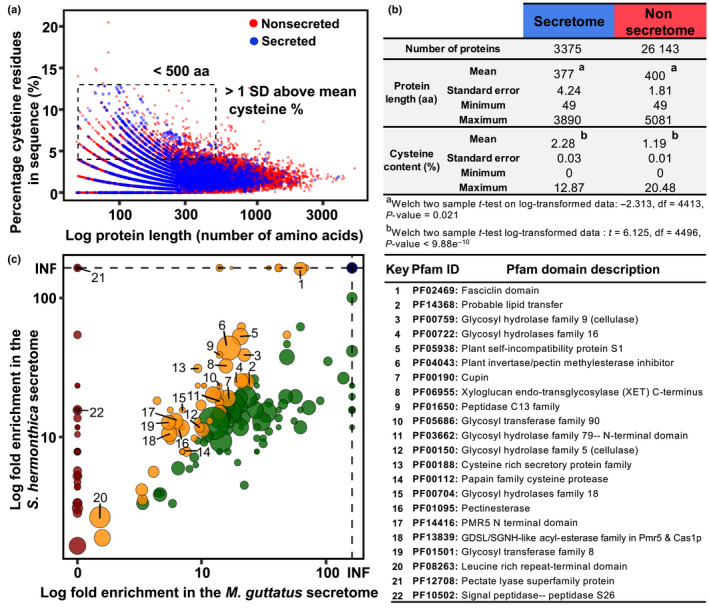
*Striga hermonthica* secretome. (a) Relationship between protein length (log scale) and cysteine content (as a % of total amino acid number) for putatively secreted (blue) and nonsecreted (red) proteins in the *S. hermonthica* proteome. Secreted proteins < 500 amino acids in length and with a cysteine % > 1 standard deviation above the mean, were selected as a subset of small, cysteine‐rich proteins. (b) Descriptive statistics for length and cysteine content for secreted and nonsecreted proteins. (c) Pfam domains enrichment (log fold change) in the *S. hermonthica* secretome, relative to the proteome as a whole, compared with the corresponding enrichment in the *Mimulus guttatus* secretome. INF denotes infinite enrichment (Pfam domain only found in the secretome). Points above the 1 : 1 diagonal were enriched more in the *S. hermonthica* secretome relative to *M. guttatus* and have been coloured accordingly. Red symbol, domains only enriched in the *S. hermonthica* secretome; yellow symbol, domains enriched more in the *S. hermonthica* secretome than in the *M. guttatus* secretome; green symbol, domains enriched more in the *M. guttatus* secretome than in the *S. hermonthica* secretome; blue symbol, domains present only in the secretome in both species. Sizes of the points were weighted according to the frequency of occurrence of each Pfam domain in the *S. hermonthica* secretome. Annotations for the most significantly enriched of the Pfam domains (*P* < 0.01) that were also enriched more in the *S. hermonthica* secretome relative to the *M. guttatus* secretome, are given in the accompanying table with their functional descriptions.

One way that parasite proteins can interact with host biology is through parasite‐directed secretion. We identified 3375 putatively secreted proteins in *S. hermonthica* (11.4% of the proteome) (Fig. S1), many of which were homologous to *A. thaliana* secreted proteins (Table S8), providing experimental evidence for secretion into the extracellular space. On average, the *S. hermonthica* secreted proteins were both significantly smaller and had a higher percentage of cysteine residues compared with the rest of the proteome (Fig. 5a,b). Genes encoding secreted proteins tended to be more clustered (within 15 kbp of their nearest neighbour) compared with all genes in the genome (*P* < 10^−4^, 10^5^ permutations) (Fig. S4) suggesting that they are likely to be arrayed in tandem and belong to large gene families (Elizondo *et al*., 2009). Functionally, the secretome was rich in protein domains involved in cell wall modification (e.g. endoglucanases, cellulases, pectin esterases, expansins, and pectate lyases), protease activity (e.g. papain‐like cysteine proteases, aspartic proteases, and subtilase proteases) and oxidoreductase activity (peroxidases, copper oxidases, and cytochrome p450 proteins) (Figs 5c, S5, S6). Three highly abundant protein domains in the secretome were described as copper oxidases (Fig. S5) and are commonly found in laccases that are involved in the generation or breakdown of phenolic components, such as lignin (Kwiatos *et al*., 2015). Small cysteine‐rich proteins are common characteristics of VFs from a range of phytoparasites (Saunders *et al*., 2012; Lu & Edwards, 2016). In *S. hermonthica*, 183 such proteins were identified (Fig. 5a) and were similar to proteins annotated as carbohydrate binding X8 domain‐containing proteins, protease inhibitor/lipid transfer proteins, PAR1‐like proteins, pectinesterases, RALF‐like proteins and thaumatin‐like proteins (Fig. S6), many of which are likely to play a role in host–*Striga* interactions (Yang *et al*., 2015; Yoshida *et al*., 2019).

We identified several protein domains in the *S. hermonthica* secretome that were enriched to a higher degree than observed in the secretome of the closely related nonparasitic plant, *M. guttatus* (Dataset S1; Fig. 5c), suggesting these functions are relevant to the parasitic lifestyle. Many of these were carbohydrate‐active enzymes (CAZymes). The xyloglucan endotransglycosylase (PF06955) domain, for example, was found in 17 *S. hermonthica* proteins (Figs 5c, S6). Xyloglucan endotransglucosylases/hydrolases (XETs) have the potential to modify either the parasite or host cell walls (or both) during parasitism (Olsen & Krause, 2017). XETs are secreted from the haustoria of the parasitic plant *Cuscuta reflexa* during a susceptible interaction on its host *Pelargonium zonale*, contributing towards pathogenicity (Olsen & Krause, 2017). Pectate lyase superfamily (PF12708) and pectinesterase (PF01095) domains were enriched in the secretome of *S. hermonthica* compared with *M. guttatus* and may act as VFs to modify host, or parasite, pectin during penetration. We found a battery of different carbohydrate‐active glycosyl hydrolase (GH) domains that were enriched in the *S. hermonthica* secretome (Figs 5c, S5). Eight *S. hermonthica* proteins were annotated as cellulases of the GH5 family (containing domain PF00150) (Fig. S6). The degradation of cellulosic β‐1,4‐glucans has been observed in susceptible sorghum roots infected by *S. hermonthica* (Olivier *et al*., 1991) and may be mediated by these secreted enzymes to facilitate the migration of *S. hermonthica* intrusive cells between host root cortical cells.

### Population genomic analysis to identify candidate virulence loci

**Fig. 6:**
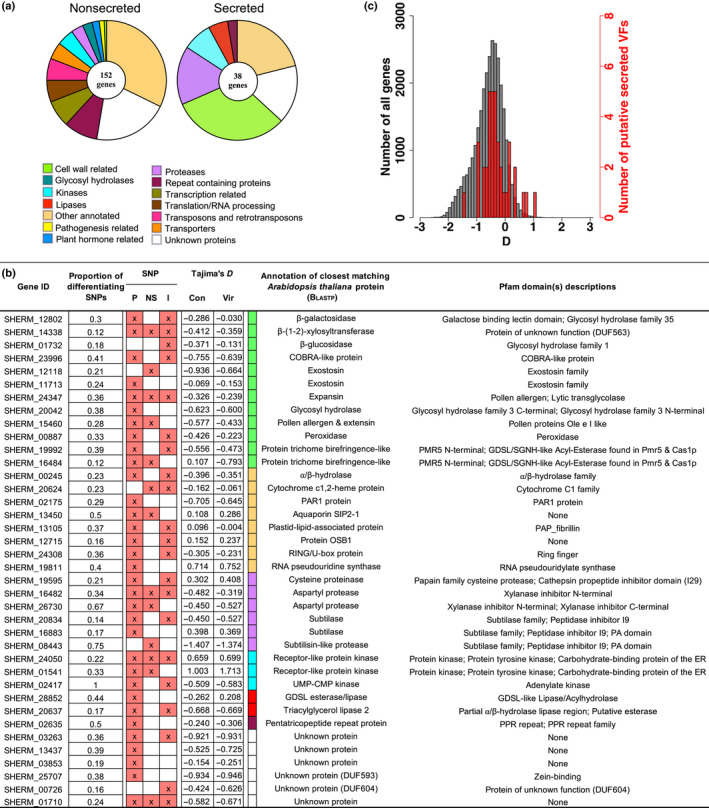
Identification of *Striga hermonthica* genes that display significant allele frequency differences between pools of individuals parasitising the susceptible rice variety (NERICA 7) and those that successfully parasitise the resistant rice variety (NERICA 17). (a) Functional categorisation of nonsecreted proteins and secreted, candidate virulence factors (VFs). (b) The 38 genes encoding putative secreted *S. hermonthica* proteins with their associated measure of differentiation (proportion of differentiating single nucleotide polymorphisms (SNPs) within the significant window) between the control and virulent sets of pools. The presence of SNPs in the promoter region (P), nonsynonymous SNPs in the coding region (NS) and those in the intronic regions (I) are indicated with an X. The annotation of the closest matching *Arabidopsis thaliana* protein is shown along with coloured boxes that correspond to the functional category assigned in the pie chart in (a). Tajima's *D* was calculated for individuals grown on NERICA 7 (Con) or NERICA 17 (Vir). (c) Comparison of Tajima's *D* for the 38 putative VFs (red) and all the genes in the genome (grey) for the control pools.

Our experimental system allowed us to identify a subset of VFs with genetic variation relevant to the ability to infect some host genotypes and not others. Hundreds of *S. hermonthica* individuals were harvested from either a resistant (NERICA‐17) or susceptible (NERICA‐7) rice cultivar, and pools of these individuals were subjected to genome re‐sequencing. After aligning the reads to our reference genome, we detected 1.8 million SNPs in genic regions. These genic regions were split into 150 741 1 kbp windows and, of these, 194 (0.13%) contained SNPs with large and consistent allele frequency differences between the bulked pools of *S. hermonthica* selected on the resistant vs the susceptible hosts (Fig. S7; Dataset S2). These highly differentiated windows were located in 190 genes and potentially encode VFs with allelic variants, influencing either structure or expression, that contribute to the ability of some individuals to parasitise NERICA‐17. Of these candidate VFs, 152 were not predicted to be secreted and were assigned to a wide range of functional categories, including putative transcription factors, hormone signalling pathways, transporters, repeat‐containing proteins and some proteins of unknown function (Dataset S2; Fig. 6a). One‐sixth (24) of these nonsecreted proteins had sequence similarity to proteins in the pathogen–host interaction database (Winnenburg *et al*., 2007). These included *S. hermonthica* proteins with sequence similarity to a putative LRR protein from *Ralstonia solanacearum*, a mitogen‐activated protein kinase from *Ustilago maydis*, a calreticulin‐like protein from *Magnaporthe oryzae* and a cytochrome P450 from *Bursaphelenchus xylophilus* (Dataset S2).

The remaining 38 genes were members of the *S. hermonthica* secretome and represented particularly strong candidate VFs associated with the ability to parasitise NERICA‐17 successfully (Dataset S2; Fig. 6a,b). These genes were categorised into six functional groups, the largest of which contained 12 genes associated with cell wall modification (Fig. 6a,b), including genes encoding an expansin protein, a COBRA‐like protein, a β‐(1–2)‐xylosyltransferase, two trichome birefringence‐like (TBL) proteins, a pollen Ole e allergen and two exostosin family proteins, all of which can function to modify the extensibility or other mechanical properties of plant cell walls (Li, 2003; Honaas *et al*., 2013; Mitsumasu *et al*., 2015) (Fig. 6b). Groups of genes annotated as proteases (six genes including subtilases, aspartyl proteases, and a cysteine proteinase), lipases (three genes) and kinases (three genes) were also found. The proteases were always associated with an inhibitor protein domain (Fig. 6b). For example, the putative aspartyl proteases possessed one or more xylanase inhibitor domain(s) (Fig. 6b). There were also eight genes encoding proteins with a range of putative functions, including a PAR1‐like protein, a probable aquaporin, an α/β‐hydrolase and two receptor‐like protein kinases (Fig. 6b). In addition, a further six genes were annotated as proteins of unknown function (Fig. 6b).

**Fig. 7:**
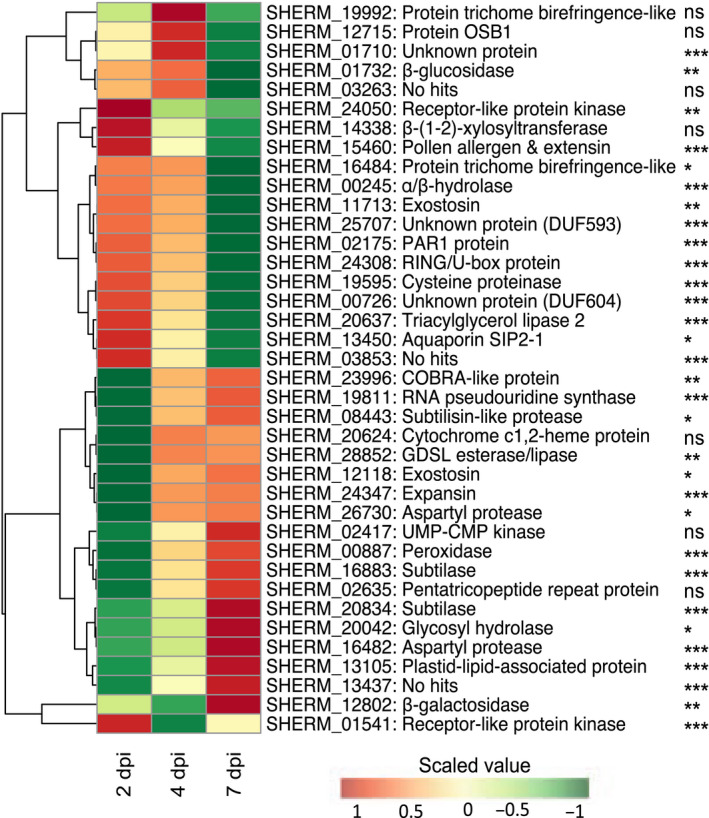
Clustered gene expression profiles of the 38 candidate, secreted virulence factors (VFs) in *Striga hermonthica* haustoria parasitising NERICA 7 at 2, 4 and 7 d post inoculation (dpi). Log_2_ fold change in expression is shown relative to expression levels in haustoria induced *in vitro*. The gene IDs and putative functions based on best Blastp hit against the *Arabidopsis thaliana* proteome correspond to Fig. 6(b). Significant changes in gene expression in haustoria during the infection time course are shown: ***, *P* < 0.001; **, *P* < 0.01; *, *P* < 0.05; ns, nonsignificant (ANOVA).

The 38 candidate VFs were investigated in more detail by quantifying changes in gene expression in haustoria at critical stages of parasite development on the susceptible rice variety NERICA‐7 by inspecting the distribution of SNPs throughout the promoter and genic regions, and testing for signatures of historical selection. Gene expression was measured in an independent experiment (Fig. 7). Changes in gene expression of attached haustoria were measured relative to gene expression in haustoria generated *in vitro*. At 2 d after inoculation of the host root, parasite haustoria were attached and parasite intrusive cells had penetrated into the host root cortex. By day 4, the parasite intrusive cells had penetrated between the endodermal cells and by day 7 had formed connections with the xylem vessels of the host, providing direct access to host resources (Fig. 1a iii).

Before attachment to the host, some of the genes encoding candidate VFs were not expressed in haustoria (e.g. subtilase gene (SHERM_16883) and subtilisin‐like protease (SHERM_08443)) or were expressed at very low levels (e.g. the peroxidase (SHERM_00887), glycosyl hydrolase (SHERM_(20042), both aspartyl proteases (SHERM_16482 and SHERM_26730) and an unknown protein (SHERM_03853)) (Dataset S3). However, all 38 genes were expressed in haustoria during the early stages of infection of the susceptible host, NERICA‐7 (Dataset S3; Fig. 7). There were two main patterns of gene expression. Firstly, 21 genes, including those mentioned above, had low levels of expression in haustoria 2 dpi, followed by an increase in expression as infection progressed (Dataset S3; Fig. 7). By contrast, 17 genes were highly expressed in haustoria 2 dpi and expression then decreased progressively with time, for example genes encoding β‐glucosidase, β‐(1–2)‐xylosyltransferase, and TBL protein SHERM_06484, all of which modify cell walls. The cysteine protease, PAR1, α/β‐hydrolase and aquaporin genes also exhibited a similar expression profile (Dataset S3; Fig. 7).

Most of the 38 genes had significantly differentiating SNPs in their promoter regions (from the start site to 2 kbp upstream). Some of these SNPs may lead to a change in the regulation of gene expression (Fig. 6b). Some genes, for example, the gene encoding the pollen Ole e allergen protein (SHERM_15460), one of the exostosin family proteins SHERM_12118), a probable aquaporin SIP2‐1 (SHERM_13450) and one of the two protein TBL genes (SHERM_16484), also had nonsynonymous SNPs in the coding region (Fig. 6b) that may result in functional differences between the alleles of these genes in individuals infecting NERICA‐7 and NERICA‐17. Finally, SNPs were also found within predicted intron regions in many of the genes (Fig. 6b).

The co‐evolutionary interactions between hosts and parasites can generate balancing selection (Frank, 1993). We predicted that genes contributing to virulence would tend to have a history of balancing selection because of the diverse range of hosts used by *S. hermonthica*. To test this prediction, we compared Tajima's *D* between candidate loci and the rest of the genome, expecting to see more positive values (Charlesworth, 2006). We used the pools from the susceptible host for this comparison because they represented the *Striga* population as a whole. As predicted, the 152 candidate loci in the *S. hermonthica* proteome (Fig. S8) and the 38 candidate loci in the secretome (Fig. 6c) had significantly elevated Tajima's *D*, on average, compared with all the genes in the genome (*P* < 0.0001 and *P* < 0.0003, respectively; 10^5^ permutations). Some loci had particularly high Tajima's *D* values, for example the two receptor‐like protein kinases (Fig. 6b). Interestingly, some loci showed large differences in Tajima's *D* between the control and virulent *S. hermonthica* pools with the largest difference seen for the TBL gene (SHERM_16484) with a negative Δ*D* (*D*_Vir_ − *D*_Con_) of −0.9. This suggests strong selection resulting in one common haplotype in the virulent pools in contrast with two or more haplotypes at intermediate frequencies in the control pools. There were also large positive Δ*D* values: 0.71, 0.16 and 0.20 for one of the putative receptor‐like protein kinases SHERM_01541, one of the aspartyl proteases, SHERM_16482, and the peroxidase SHERM_00887, respectively. This suggests that a rare haplotype in the control pools is present at intermediate frequency in the virulent pools. Overall, these changes indicate that selection on the resistant host caused changes in frequency of multi‐SNP haplotypes at these loci, haplotypes that may have been created by areas of low recombination or by recent invasion of new variants under positive selection (Cutter & Payseur, 2013) and which underlie the ability of some *S. hermonthica* individuals to overcome resistance in NERICA‐17.

## Discussion

In parasitic plants such as *S. hermonthica*, a subset of secreted proteins is likely to function as VFs and contributes towards parasite fitness by facilitating host colonisation. We combined *in silico* prediction of the *S. hermonthica* secretome and pooled sequencing of parasites derived from susceptible and resistant rice hosts, both facilitated by the first available *S. hermonthica* genome assembly, to discover potential VFs. Our candidate VFs had very different allele frequencies between replicated pools derived from susceptible and resistant hosts, suggesting strong selection for particular variants that facilitate successful colonisation despite host resistance. They encompass a wide range of different functional categories.

### Candidate VFs point to key functions associated with pathogenicity

The largest proportion of our 38 candidate secreted VFs (with the largest allele frequency differences) exhibited functions relating to cell wall modification. Cell wall modification is a critical step in plant invasions by many different parasites, including *S. hermonthica*. Upon host root contact, *Striga* epidermal cells differentiate to form elongated intrusive cells (Musselman & Dickison, 1975) which intrude between the host cell walls of the host epidermis, cortex, casparian strip and endodermal barrier, to reach the host xylem vessels and establish a xylem bridge (Cui *et al*., 2016; Wakatake *et al*., 2018), therefore allowing access to host water and nutrients (Yoshida *et al*., 2016; Clarke *et al*., 2019). Consistent with this, our candidate’s secreted VFs included an expansin, pollen allergen‐like proteins, exostosins, a β‐glucosidase, a glycosyl family protein 3 (likely to be a β‐glucosidase or β‐xylosidase), a β (1–2) xylosyltransferase, a peroxidase and two TBL proteins, all of which may function to modify or degrade different components of the cell walls.

In our study, the TBL protein, SHERM_16484, showed the highest difference in Tajima's *D* between the control and virulent pools, consistent with selection favouring one haplotype on the resistant NERICA‐17, out of several haplotypes present in the population. This gene contained SNPs in the promoter region and nonsynonymous SNPs in the coding region. In *A. thaliana* and *O. sativa*, TBL proteins belong to large gene families with functions related to cell wall modifications. At‐TBL44 has been implicated in pectin esterification (Vogel *et al*., 2004; Bacete *et al*., 2018), whilst in rice other members of this family appear to be involved in acetylation of xylan moieties in cell walls (Gao *et al*., 2017). In each case, changes in enzyme activity altered resistance in *A. thaliana* to powdery mildew and in rice to leaf blight (Vogel *et al*., 2004; Gao *et al*., 2017).

Our study supports the growing body of evidence that the production of cell wall modifying and degrading enzymes represents a general strategy used by parasitic plants to facilitate successful invasion of the host (Honaas *et al*., 2013; Mitsumasu *et al*., 2015; Yang *et al*., 2015), or alter the composition of their own cell walls to protect against autodegradation (Johnsen *et al*., 2015). A comparative transcriptome study of *S. hermonthica*, *Tryphasaria versicolor* and *Phelipanche aegyptiaca* identified a core set of *c*. 180 genes that were upregulated in parasite haustoria following attachment and penetration of their hosts (Yang *et al*., 2015). This set was significantly enriched for cell wall and extracellular localisation annotation terms. Johnsen *et al*. (2015) compared differences in carbohydrate epitopes in cross‐sections of *Pelargonium zonale* parasitised by *C. reflexa* together with an analysis of enzymes within haustoria and concluded that it was likely that the parasite secretes some enzymes that remodel its own cell walls for protection.

Several candidate VFs were predicted to have protease activity. Interestingly, all had a dual‐domain predicted structure consisting of a propeptide inhibitor domain and a catalytic protease domain. In other such protease enzymes, the propeptide domain autoinhibits the enzyme activity until cleavage of this inhibitor domain activates the catalytic domain (Shindo & Van Der Hoorn, 2007). This provides a mechanism by which the parasite could initially secrete an inactive VF that only becomes active once in the host environment. A similar dual‐domain structure was found for a highly expressed, haustorium‐specific cysteine protease, cuscutain, in the shoot‐parasitic plant *C. reflexa* (Bleischwitz *et al*., 2010). The main cuscutain protein was targeted to the extracellular space by the prepeptide and deletion of the inhibitor propeptide subunit converted the enzyme to an active form, which positively contributed towards pathogenicity via protein degradation (Bleischwitz *et al*., 2010). These authors also hypothesised that the large amount of pectin on the surface of *C. reflexa* haustoria may protect parasite tissue from degradation.

Subtiliases perform diverse functions in plants including protein turnover, plant development and biotic and abiotic interactions (Figueiredo *et al*., 2018). In our study the three subtilases were highly upregulated from 4 to 7 dpi in the susceptible host. The expression of subtilases was also upregulated in haustoria of *S. asiatica* (Yoshida *et al*., 2019) and *Phtheirospermum japonicum* (Ishida *et al*., 2016) during infection of their respective host plants. A transcriptome analysis of laser dissected intrusive cells of *P. japonicum* has recently revealed that four subtilases, only found in parasitic plants, were highly expressed from 3 to 7 dpi and that inhibition of the activity of these subtilases delayed the maturation of the haustorium and xylem bridge formation, consistent with an important role in parasitism (Ogawa *et al*., 2021). It is interesting to note that one of the most common phenotypes of resistance in NERICA‐17 is the inability to form a xylem bridge with the host (Fig. 1a vi).

In plants, receptor‐like kinases are a large gene family that have multiple functions in regulating plant growth, development and immunity (Lin *et al*., 2013). Two candidate VFs were annotated as receptor‐like protein kinases, one of which, SHERM_01541, had a large positive Δ*D* value which suggests this haplotype may be present at a greater frequency in the virulent compared with susceptible *Striga* pools. Both genes were upregulated in *S. hermonthica* haustoria from 4 to 7 dpi. Although the function of these genes has however to be determined, Yang *et al*. (2015) also observed upregulation of genes encoding receptor‐like protein kinases in *S. hermonthica* during haustorial development. Some of our VFs had predicted functions for which a role in virulence is less clear, including a putative aquaporin, PAR1 protein and a cytochrome P450. However genes with similar functional annotations were also identified through comparative transcriptomics approaches as likely to be important in parasitism in parasitic Orobanchaceae species (Yang *et al*., 2015), highlighting the robustness of this approach and providing exciting avenues for further investigation.

### Conclusions and the way forward

*Striga hermonthica* parasitises many different host species and varieties, often within the same geographical area. Populations therefore encounter many different forms of resistance, which they experience as a highly heterogeneous environment. This is expected to maintain genetic diversity at many loci contributing to virulence, consistent with observations from field studies that resistant varieties, of any particular crop species, are often parasitised by one or two *S. hermonthica* individuals (Gurney *et al*., 2006; Cissoko *et al*., 2011; Rodenburg *et al*., 2015, 2017). A typical example is the host–parasite combination used in this study, in which NERICA‐17 is strongly resistant to the *S. hermonthica* population from Kibos, with just a few individuals forming successful attachments, whereas NERICA‐7 is extremely susceptible.

This host range predicts a wide range of functions implicated in overcoming host resistance. We detected 190 candidates (secreted and nonsecreted) for contribution to virulence, with large allele frequency differences between our control and virulent pools, including many gene families. It is likely that many additional candidate VFs would be revealed by repeating this comparison on other resistant hosts. An important question for the future will be to determine how individual VFs (and their allelic variants) are implicated in overcoming resistance for specific hosts or across a range of hosts. Ideally this requires a high‐throughput, efficient, transformation system for *S. hermonthica*. Although it is now possible to produce and transform *S. hermonthica* callus *in vitro* (Waweru *et al*., 2019), to the best of our knowledge it is not however possible to regenerate plants.

The wide host range also predicts the maintenance of variation at virulence loci by balancing selection. We found the overall Tajima's *D* in *S. hermonthica* to be negative, perhaps reflecting population expansion following the spread of agriculture, but our candidate loci had significantly higher Tajima's *D* on average, consistent with balancing selection on these loci maintaining multiple alleles. Further understanding the maintenance of variation at virulence loci will be critical to managing the evolution of virulence as part of a sustainable control strategy (Mikaberidze *et al*., 2015).

Effective control of *S. hermonthica* is essential for food security and poverty alleviation for small‐holder subsistence farmers. The use of resistance crop varieties is recognised as sustainable and cost effective (Scholes & Press, 2008), but requires a knowledge of the VFs involved, their allelic variation within and between *Striga* populations and their interaction with different host resistance alleles. Our experimental approach and identification of candidate VFs and allelic variation within a *S. hermonthica* population, is a critical first step in this direction. This approach has not been applied previously to investigate the virulence of any parasitic plant. Its success here paves the way to apply similar methods to other host–parasite combinations, therefore underpinning the development of sustainable control strategies.

## Author contributions

JDS and RKB planned and designed the research. SQ, PZ and JDS contributed to the production of *S. hermonthica* materials and extraction of DNA for genome and pooled sequencing. MB carried out library preparation and sequencing of the *S. hermonthica* genome. SQ led the genome assembly and annotation with contributions from JMB, RC, JDS and RKB. JMB carried out the prediction and analysis of the *S. hermonthica* secretome. SQ mapped the pooled *S. hermonthica* sequence reads to the *S. hermonthica* genome. SQ, RKB and JMB contributed to the population genomic analyses. JMB, PZ and JDS contributed to the analysis of changes in gene expression in *S. hermonthica* haustoria. All authors contributed to writing of the manuscript. SQ and JMB contributed equally to this work.

## Supporting information


**Dataset S1** Pfam domains enriched in the secretome of *Striga hermonthica* and *Mimulus guttatus*.

**Dataset S2** Genes encoding putative secreted and nonsecreted *Striga hermonthica* virulence factors.

**Dataset S3** FPKM values for *Striga hermonthica* haustoria during infection of the susceptible rice variety NERICA 7.

**Fig. S1** Three‐step pipeline to predict the *Striga hermonthica* secretome and subsets of candidate pathogenicity‐related genes.
**Fig. S2** Distribution of mean ΔAICcv difference to the maximum ΔAICcv ratios in each distance interval.
**Fig. S3** Comparison of a maximum likelihood tree constructed in MegaX and a species tree generated in OrthoFinder.
**Fig. S4** Testing gene clustering in the secretome of *Striga hermonthica*.
**Fig. S5** Relative abundance of Pfam domains in the *Striga hermonthica* secretome or in the rest of the proteome.
**Fig. S6** Functional categorisation of four subsets of proteins selected from the *Striga hermonthica* secretome.
**Fig. S7** Mean ΔAICcv in relation to the numbers of SNPs in 1 kb windows in genic regions.
**Fig. S8** Comparison of Tajima's *D* for the 152 putative virulence factors and all the genes in the genome for the control pools.
**Methods S1** Detailed list of methods and supplementary references.

**Notes S1** JAVA‐script for testing gene clustering in the secretome.

**Notes S2** R script for the permutation test on mean ΔAICcv values.

**Notes S3** JAVA‐script for obtaining the permutation *P*‐values on mean ΔAICcv values of each 1 kb window.

**Table S1** Sequencing information for the *Striga hermonthica* reference genome and the bulked samples for pooled re‐sequencing analysis.
**Table S2** Plant species included in the analysis of genome size, heterozygosity and repetitiveness.
**Table S3** Summary statistics for the *Striga hermonthica* genome assembly.
**Table S4** Repeat elements identified in the *Striga hermonthica* genome.
**Table S5** Comparison of the *Striga hermonthica* genome annotation with other plant species.
**Table S6** Busco completeness analysis using 2326 core orthologous genes for eudicots (version: eudicots_odb10).
**Table S7** Enriched GO terms associated with Buscos that were missing from the genomes of only the two *Striga* species, only the two *Cuscuta* species or both the two *Striga* species and the two *Cuscuta* species.
**Table S8** Predicted subcellular location of *Striga hermonthica* proteins according to their closest orthologue in *Arabidopsis thaliana*.
Please note: Wiley Blackwell are not responsible for the content or functionality of any Supporting Information supplied by the authors. Any queries (other than missing material) should be directed to the *New Phytologist* Central Office.

## Data Availability

Raw reads for the pooled *S. hermonthica* sequences and for the *S. hermonthica* genome sequence, the assembled genome sequence and annotations have been submitted to the European Nucleotide Archive (ENA) browser at http://www.ebi.ac.uk/ena/data/view/ under the Project ID: PRJEB35606 (ERP118683). JAVA and R script files for the work detailed in Methods S1 can be found in Notes [Link], [Link], [Link].
