# Supplementary material for: Genome‐enabled discovery of candidate virulence loci in Striga hermonthica, a devastating parasite of African cereal crops
Source: New Phytol. 2022 Jul 7;236(2):622–38. doi: 10.1111/nph.18305 (PMC9795911; doi:10.1111/nph.18305)
Supplement: Supplementary file 4 — Fig. S1 Three‐step pipeline to predict the Striga hermonthica secretome and subsets of candidate pathogenicity‐related genes. Fig. S2 Distribution of mean ΔAICcv difference to the maximum ΔAICcv ratios in each distance interval. Fig. S3 Comparison of a maximum likelihood tree constructed in MegaX and a species tree generated in OrthoFinder. Fig. S4 Testing gene clustering in the secretome of Striga hermonthica. Fig. S5 Relative abundance of Pfam domains in the Striga hermonthica secretome or in the rest of the proteome. Fig. S6 Functional categorisation of four subsets of proteins selected from the Striga hermonthica secretome. Fig. S7 Mean ΔAICcv in relation to the numbers of SNPs in 1 kb windows in genic regions. Fig. S8 Comparison of Tajima's D for the 152 putative virulence factors and all the genes in the genome for the control pools. Methods S1 Detailed list of methods and supplementary references. [file NPH-236-622-s005.pdf]

***New Phytologist* Supporting Information**

Article title: Genome-enabled discovery of candidate virulence loci in *Striga hermonthica*, a devastating parasite of African cereal crops

Authors: Suo Qiu, James M. Bradley, Peijun Zhang, Roy Chaudhuri, Mark Blaxter, Roger K Butlin, Julie D. Scholes

Article acceptance date: 18 May 2022

The following Supporting Information is available for this article:

Fig. S1 Three step pipeline to predict the *Striga hermonthica* secretome and subsets of candidate pathogenicity-related genes.
Fig. S2 Distribution of mean ΔAICcv difference to the maximum ΔAICcv ratios in each distance interval.
Fig. S3 Comparison of a maximum likelihood tree constructed in MEGA X and a species tree generated in OrthoFinder.
Fig. S4 Testing gene clustering in the secretome
Fig. S5 Relative abundance of Pfam domains in the *Striga hermonthica* secretome or in the rest of the proteome.
Fig. S6 Functional categorization of 4 subsets of proteins selected from the *Striga hermonthica* secretome.
Fig. S7 Mean ΔAICcv in relation to the numbers of SNPs in 1kb windows in genic regions.
Fig. S8 Comparison of Tajima's D for the 152 putative VFs (green) and all the genes in the genome for the control pools.

Table S1 Sequencing information for the *Striga hermonthica* reference genome and the bulked samples for pooled re-sequencing analysis (see separate file - Tables 1 – 8.xlsx).
Table S2 Plant species included in the analysis of genome size, heterozygosity and repetitiveness (see separate file - Tables 1 – 8.xlsx).
Table S3 Summary statistics for the *Striga hermonthica* genome assembly (see separate file - Tables 1 – 8.xlsx).
Table S4 Repeat elements identified in the *Striga hermonthica* genome (see separate file - Tables 1 – 8.xlsx).
Table S5 Comparison of the *Striga hermonthica* genome annotation with other plant species (see separate file - Tables 1 – 8.xlsx).
Table S6 BUSCO completeness analysis using 2,326 core orthologous genes for eudicots (version: eudicots_odb10) (see separate file - Tables 1 – 8.xlsx).
Table S7. Enriched GO terms associated with BUSCOs that were missing from the genomes of only the two Striga species, only the two Cuscuta species or both the two Striga species and the two Cuscuta species.
Table S8 Predicted subcellular location of *Striga hermonthica* proteins according to their closest ortholog in *Arabidopsis thaliana*. (See separate file - Tables S1 – S8.xlsx).

Methods S1 – Detailed list of methods and supplementary references

Dataset S1 Pfam domains enriched in the secretome of *Striga hermonthica* and *Mimulus guttatus* (see separate xlsx file).
Dataset S2 Genes encoding putative secreted and non-secreted *Striga hermonthica* Virulence Factors (VFs) (see separate xlsx file).
Dataset S3 FPKM values for *Striga hermonthica* haustoria during infection of the susceptible rice variety NERICA 7 (see separate xlsx file).

Note S1 JAVA-script for testing gene clustering in the secretome.
Note S2 R-script for the permutation test on mean ΔAICcv values
Note S3 JAVA-script for obtaining the permutation P values on mean ΔAICcv values of each 1Kb window (see separate files .txt files).

Fig. S1 Three step pipeline to predict the *Striga hermonthica* secretome and subsets of candidate pathogenicity-related genes. Step 1: Three SignalP algorithms were used to identify *S. hermonthica* proteins with a predicted secretion signal at their N-terminus. These proteins were then checked for a transmembrane helix in the remaining part of the protein and only retained if no transmembrane domains were identified. Step 2: A range of structural and functional information was gathered for putative secreted proteins. Step 3: the secretome was refined into sets of candidate pathogenicity-related genes using the information gleaned in step 2. In addition to the three secretome subsets, a stand-alone BLASTp analysis was carried out against the pathogen-host interaction database.

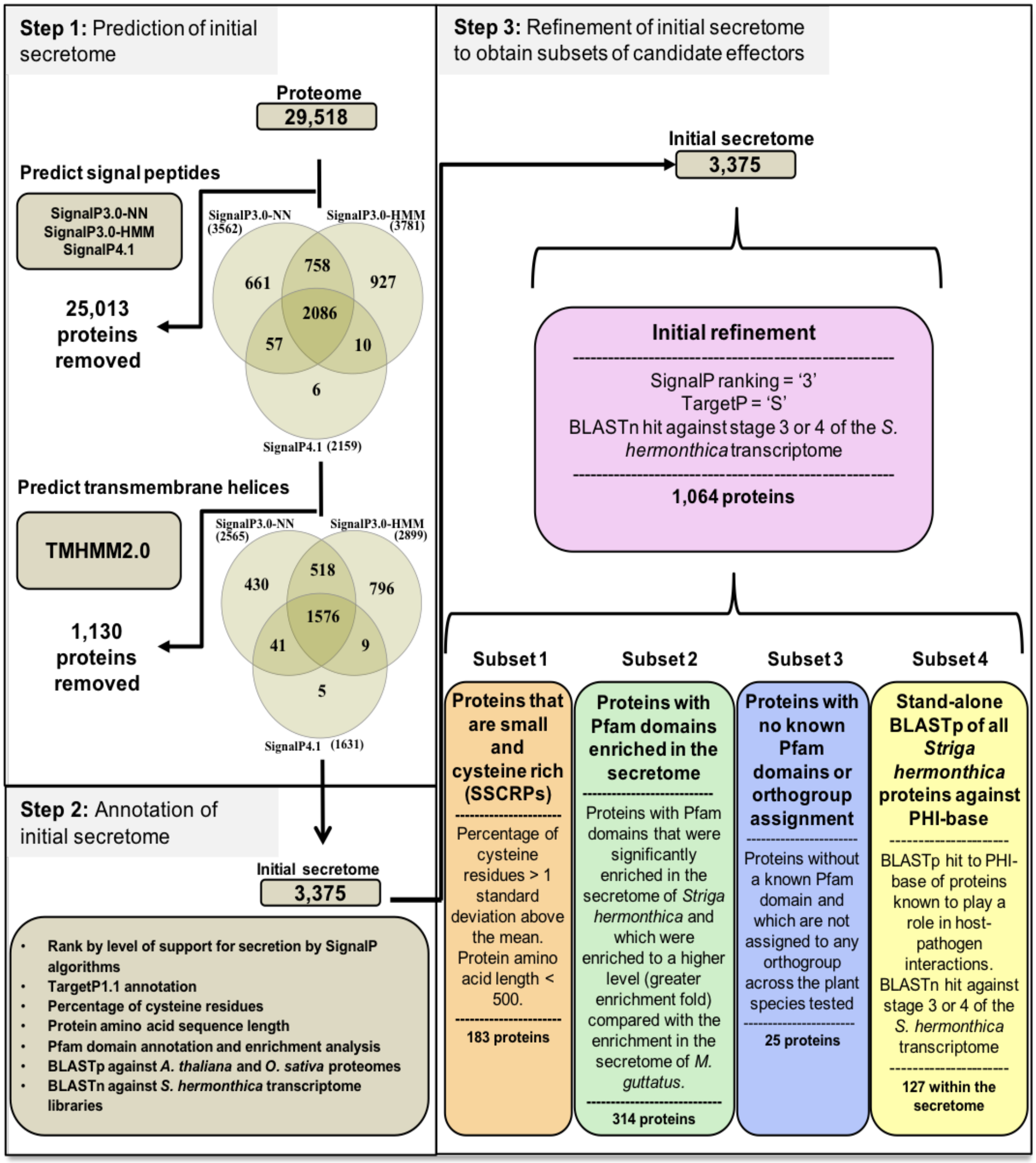

Fig. S2 Distribution of mean ΔAICcv difference to the maximum ΔAICcv ratios in each distance interval. The upper and lower limits of the boxes are the first and third quartiles of the ratios in each distance interval; medians of the data are shown as bands; means are shown in red dots; the whiskers represent the smallest and biggest datum that are still within 1.5 times interquartile range of the lower and upper quartile, and the outliers are shown as black dots.

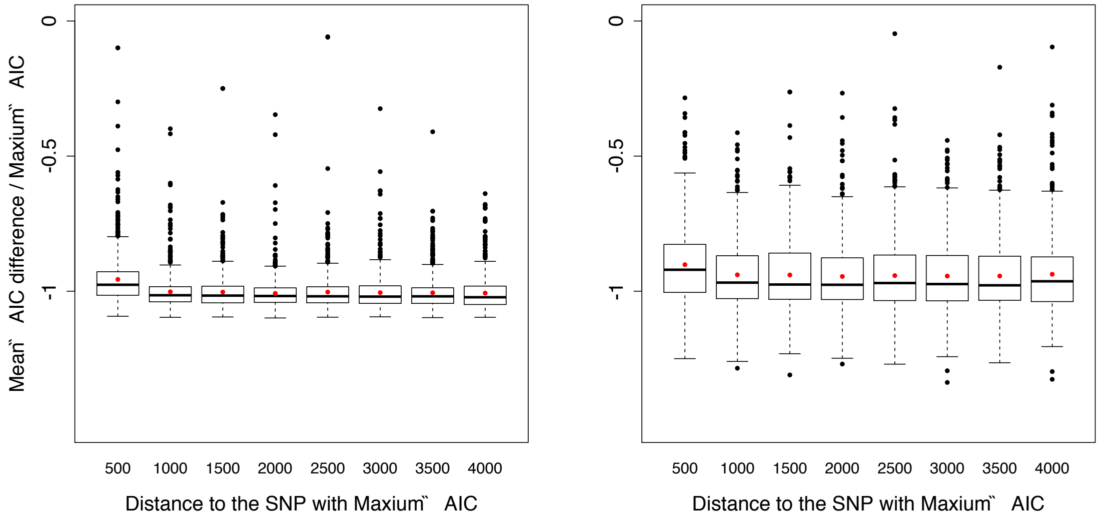

Fig. S3 Comparison of a maximum likelihood tree constructed in MEGA X and a species tree generated in OrthoFinder. (a) A maximum likelihood (ML) tree constructed in MEGA X. The evolutionary history was inferred by using the Maximum Likelihood method and the w/freq. model of Jones *et al.,* (1992). The percentage of trees in which the associated taxa clustered together in the bootstrap test (500 replicates) is shown next to the branches. Initial tree(s) for the heuristic search were obtained automatically by applying Neighbor-Join and BioNJ algorithms to a matrix of pairwise distances estimated using the JTT model, and then selecting the topology with superior log likelihood value. A discrete Gamma distribution was used to model evolutionary rate differences among sites (5 categories (+G, parameter = 0.6168)). (b) A species tree generated in OrthoFinder. The species tree was inferred from a set of unrooted orthogroup gene trees using STAG and rooted using STRIDE.

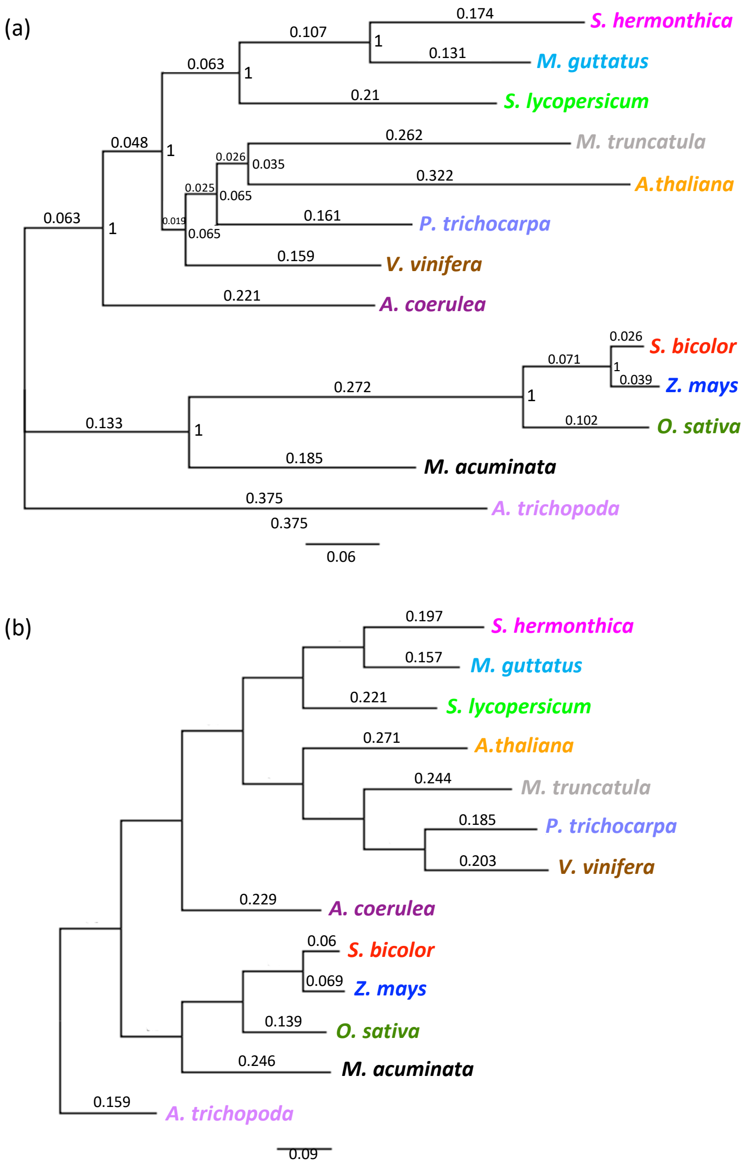

Fig. S4 Testing gene clustering in the secretome of *Striga hermonthica*. The red dot represents the number of genes in the secretome with MinDis falling into each 5Kb distance interval. The upper and lower limits of the boxes are the first and third quartiles in the 10,000 permutations in each distance interval; medians of the data are shown as bands in the boxes; the whiskers represent the smallest and biggest datum that are still within 1.5 times interquartile range of the lower and upper quartile, and the outliers are shown as black dots.

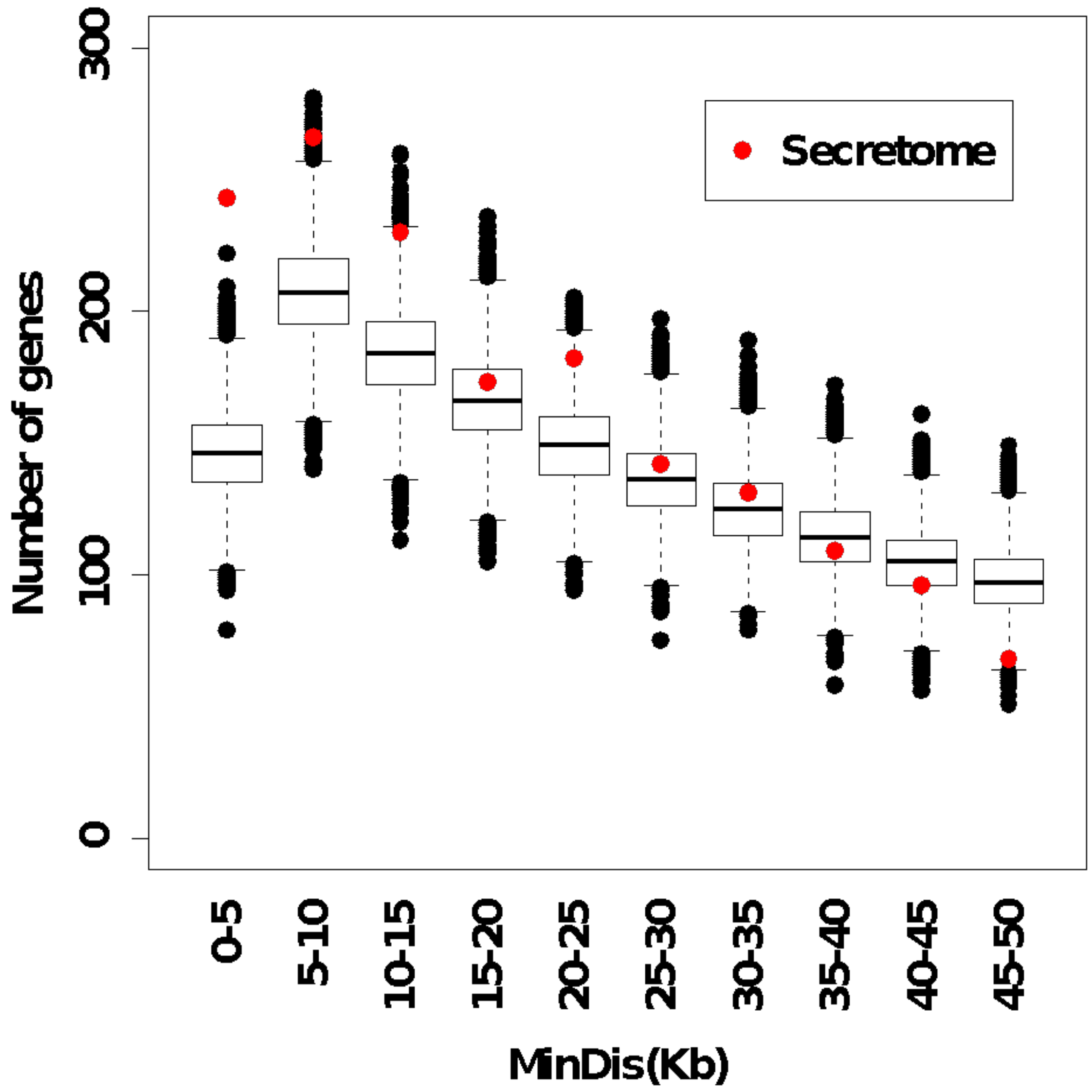

Fig. S5 Relative abundance of Pfam domains in the *Striga hermonthica* secretome or in the rest of the proteome (non-secretome). The relative abundance of each Pfam domain is plotted as a percentage of all Pfam domains found in the secretome or the non-secretome. The 45 Pfam domains with the highest difference in relative abundance between the two data sets are shown.

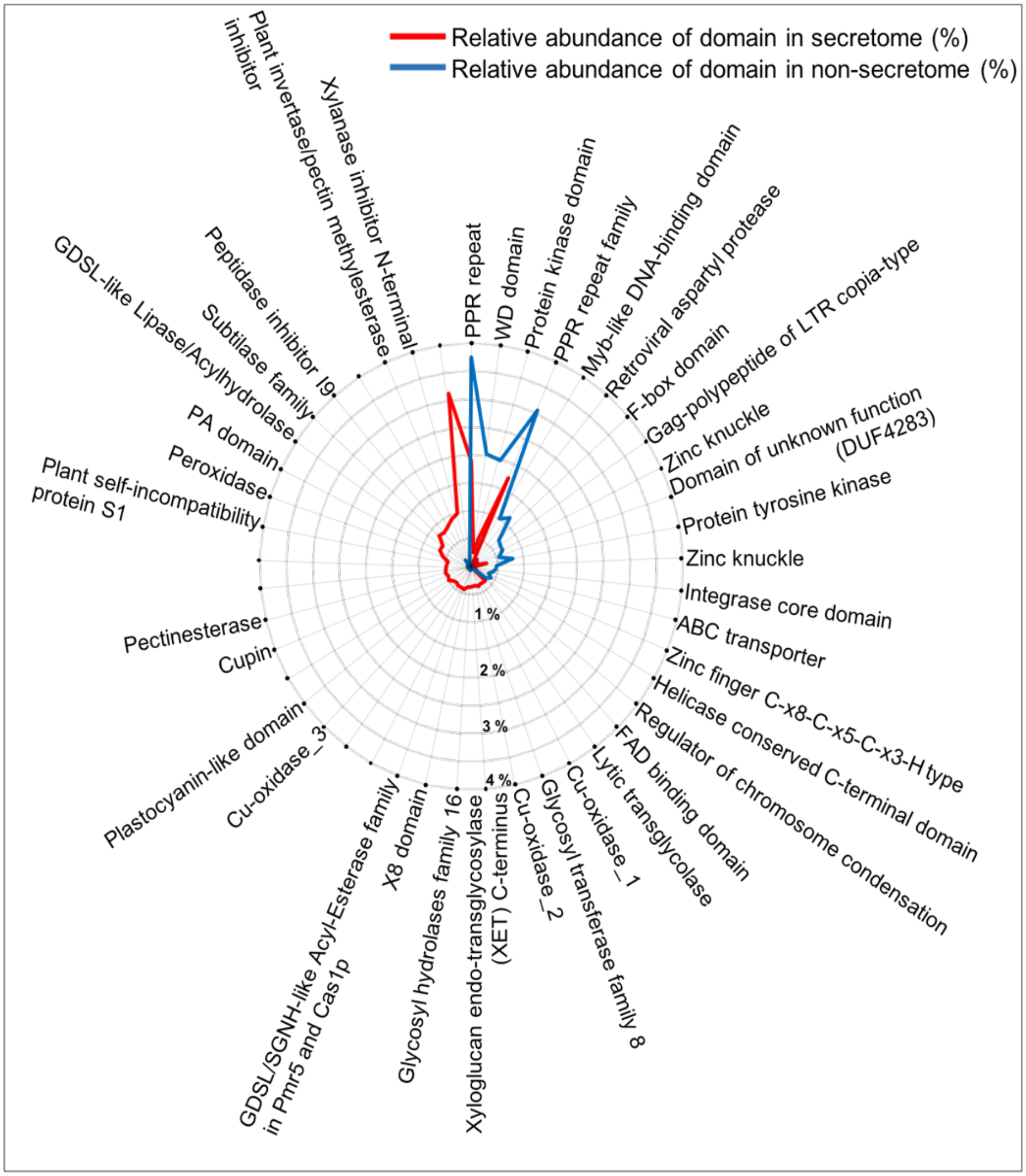

Fig. S6 Functional categorization of 4 subsets of proteins selected from the *Striga hermonthica* secretome. *S. hermonthica* proteins were assigned into functional groups based on the closest BLASTp match with *Arabidopsis thaliana*. If no clear annotation was available, the closest match with *Oryza sativa* was used. If no clear matches were obtained for either *A. thaliana* or *O. sativa,* the Pfam domains were used to infer function. Subset 1: small, cysteine rich proteins. Subset 2: proteins with Pfam domains enriched in the secretome of *S. hermonthica* to a greater degree than in the secretome of *Mimulus guttatus*. Subset 3: proteins with no known PFAM domain or orthogroup assignment. Subset 4: proteins with a BLASTp match against a protein in the pathogen-host interaction database. For subsets 1, 2 and 4, all *S. hermonthica* protein functions which were only represented once were categorized as 'other'. Subset 3 only contained 25 *S. hermonthica* proteins and therefore all protein functions were shown, even if they only occurred once. Venn diagram shows the overlap between the four subsets of secreted *S. hermonthica* proteins.

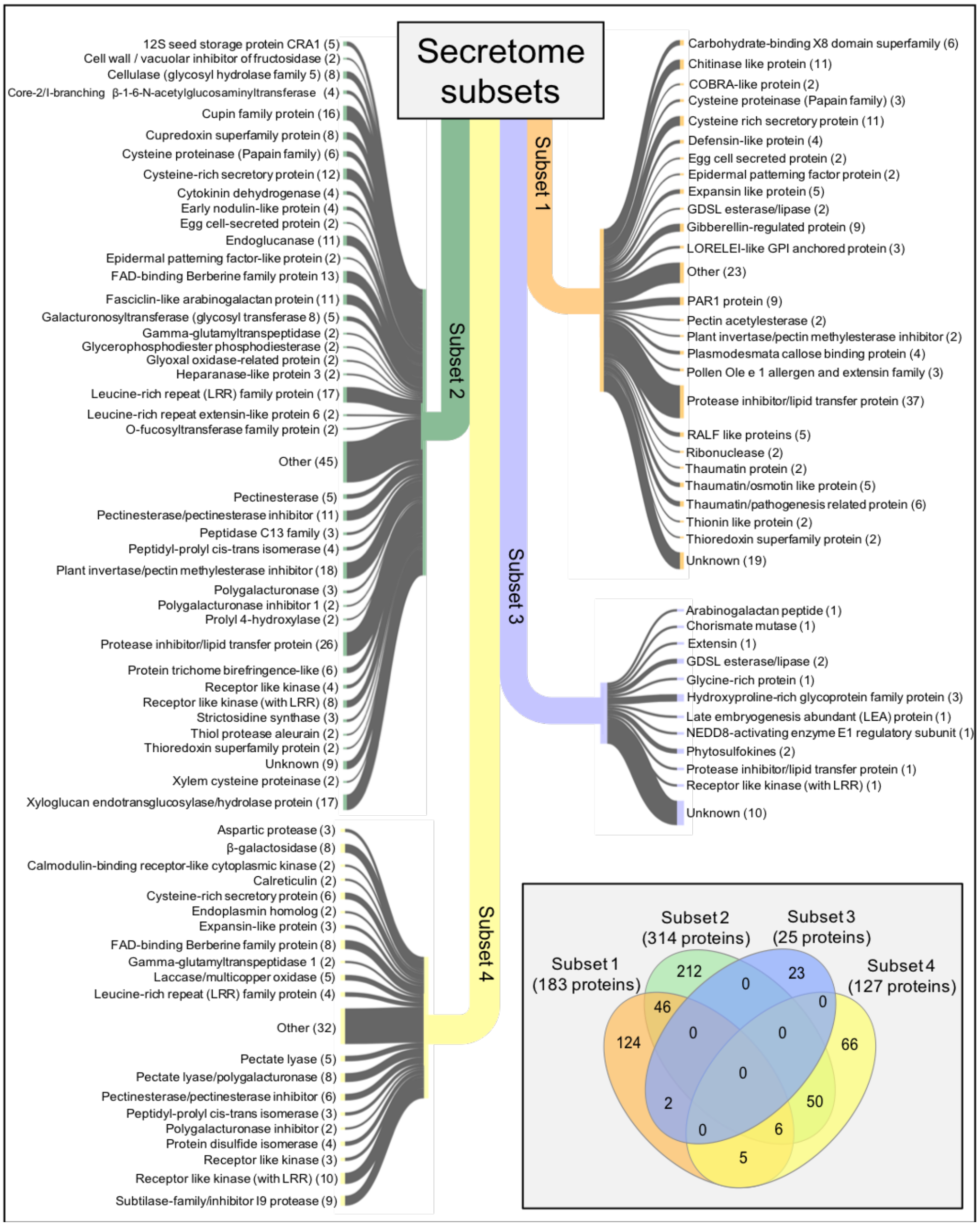

Fig. S7 Mean ΔAICcv in relation to the numbers of SNPs in 1kb windows in genic regions. ΔAICcv is a measure of the magnitude, and consistency across pools, of the allele frequency difference between *Striga hermonthica* growing on resistant NERICA 17 (virulent) and those growing on susceptible NERICA 7 (control). Significant windows in genic regions contributing to the secretome (red) and non-secretome (green) were identified by permutation. All the non-significant windows are shown in grey.

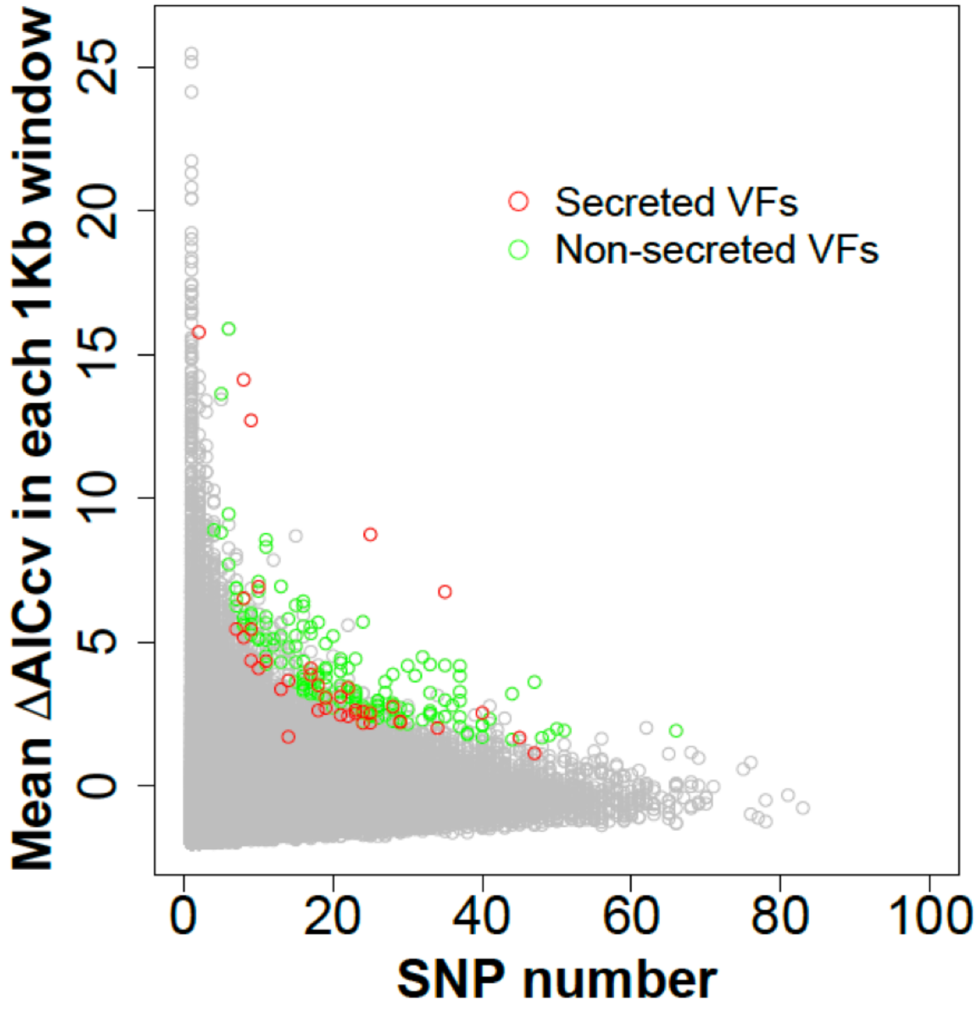

Fig. S8 Comparison of Tajima's D for the 152 putative non secreted VFs (green) and all the genes in the genome (grey) for the control pools. The 152 candidate loci in the proteome had significantly elevated D on average ($p < 0.0001$, 10^5 permutations) compared to the rest of the genome.

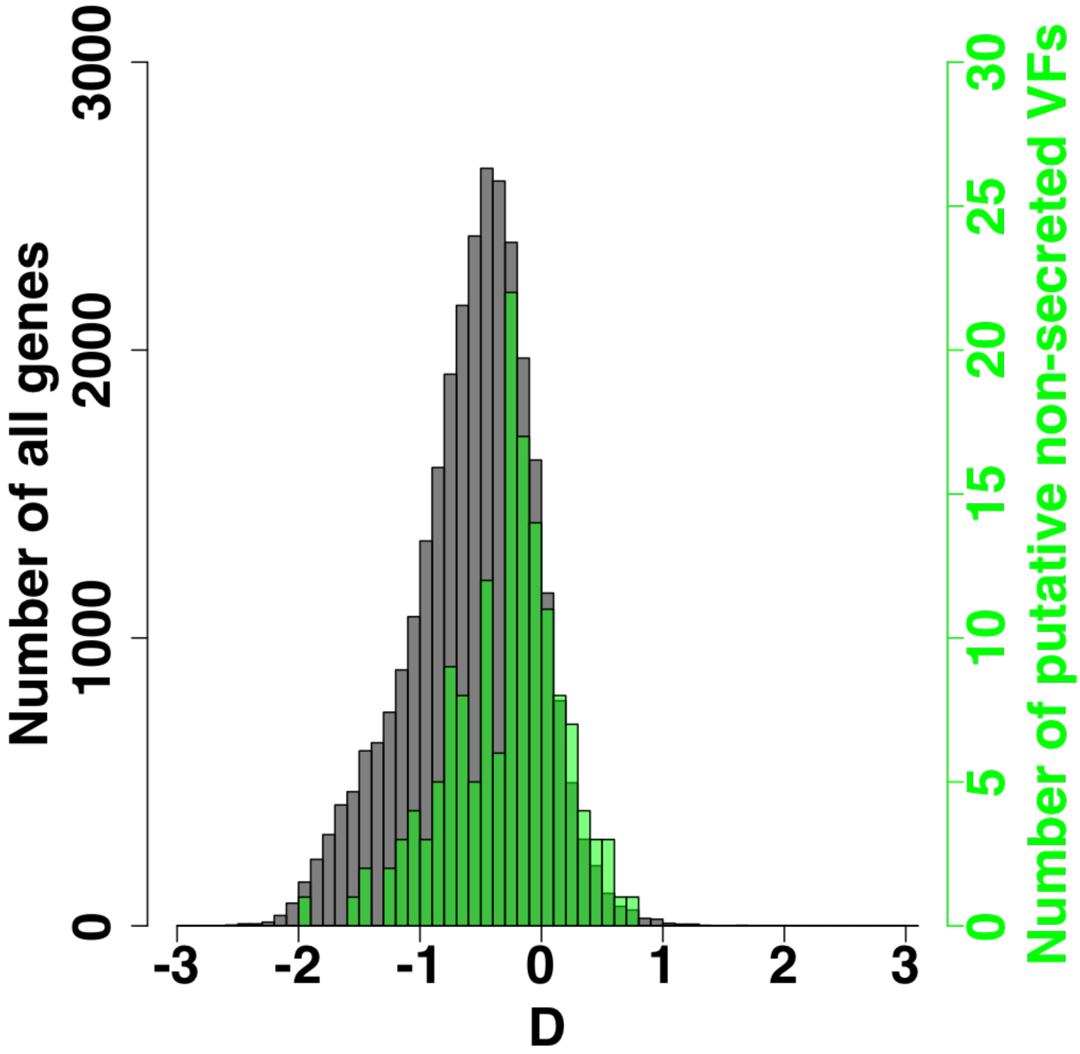

Methods S1

DNA extraction from *Striga hermonthica*

Each pool of 100 *S. hermonthica* individuals was randomly divided into 25 groups of four individuals (20 mg of tissue per individual) for DNA extraction. To remove high levels of secondary metabolites and polysaccharides in *S. hermonthica*, lysed plant tissues were first washed in a buffer containing 2 % polyvinylpyrrolidone, 0.25 M NaCl, 0.2 M Tris-HCl and 50 mM EDTA. DNA was then extracted following the CTAB protocol (Stewart & Via, 1993). The quality of DNA was checked by gel electrophoresis and quantified by Nanodrop. An equal quantity of DNA from each of the 25 samples was then combined to form one biological replicate.

De novo assembly of the *Striga hermonthica* genome

For *de novo* genome assembly, adapters and low quality reads (Q < 20) were removed using CutAdapt (Martin, 2011). Only reads longer than 50 bp for the paired-end libraries and 30 bp for the mate-pair libraries were retained for subsequent analyses. Duplicated reads generated by PCR amplification in the library construction process were removed using FASTUNIQ (Xu *et al.,* 2012). Sequencing errors, which can create difficulties for the short-read assembly, were corrected using the software BLESS, a *k*-mer spectrum-based method designed to remove *k*-mers with a small number of occurrences (Heo *et al.,* 2014*)*.

Annotation of the *S. hermonthica* genome

The genome was annotated using three methods Firstly, gene structures were inferred using a *S. hermonthica* transcriptome dataset of cDNAs collected from *S. hermonthica* individuals at eight developmental stages, generated by the Parasitic Plant Genome Project (PPGP) (Westwood *et al.,* 2012; Yang *et al.,* 2015). The reads were mapped onto the *S. hermonthica* genome assembly using TopHat to identify exon regions and splice positions (Trapnell *et al.,* 2009). Transcriptome-based gene structures were predicted using Cufflinks (Trapnell *et al.,* 2012) and candidate coding regions were then constructed in Transdecoder (https://github.com/TransDecoder/). Secondly, protein sequences from *Arabidopsis thaliana* (TAIR10), *Mimulus guttatus* (v2.0), *Solanum lycopersicum* (ITAG2.4), *Oryza sativa* (IRGSP1.0) and *Sorghum bicolor* (79), were used to determine consensus gene models in the genome. The protein sequences were mapped onto the *S. hermonthica* genome using TBLASTN and pairwise alignments were then input into Genewise (Birney 2004) to predict gene models in *S. hermonthica*. Thirdly, an *ab initio* method was used for *de novo* prediction of genes in the *S. hermonthica* genome using the software, Braker, with default settings. (Hoff *et al.,* 2016). Finally, Evidence Gene Modeler was used to integrate various gene models from these approaches (Haas *et al.,* 2008).

Inference of orthogroups (OG)

Orthologous gene groups (Orthogroups or OGs) among 13 plant species (*Amborella trichopoda*, *Musa acuminata*, *Oryza sativa*, *Zea mays*, *Sorghum bicolor*, *Aquilegia coerulea*, *Solanum lycopersicum*, *Mimulus guttatus*, *Striga hermonthica*, *Arabidopsis thaliana*, *Medicago truncatula*, *Populus trichocarpa* and *Vitis vinifera*) were inferred using the software OrthoFinder v2 (Emms & Kelly, 2015). Except for *S. hermonthica*, the proteomes of these species were downloaded from Phytozome V12.1 (https://phytozome.jgi.doe.gov/pz/portal.html). The number of genes per

species for each OG was transformed into a matrix of *Z*-scores to quantify gene family expansion / contraction in comparison with other plant genomes. *Z*-score is defined as $Z_X = (N_X - \text{mean})/SD$, where N_X is the gene count for the species X in the focal orthogroup, mean is defined as the mean gene count of the 13 species in the focal orthogroup and SD is the standard deviation of gene count of the 13 species in the orthogroup. The significance of expansion or contraction was determined using CAFE v4.2 (Han *et al.,* 2013). Specifically, multi-species alignments of 42 single-copy genes from the OrthoFinder results were first constructed using MAFFT v7.407 (Katoh & Standley, 2013). A maximum likelihood (ML) tree using the alignments of the 42 single-copy genes was constructed in MEGA X (Kumar *et al.,* 2018). The evolutionary history was inferred by using the Maximum Likelihood method and the w/freq. model of Jones *et al.,* (1992). The tree with the highest log likelihood (-163592.96) is shown. The percentage of trees in which the associated taxa clustered together in the bootstrap test (500 replicates) is shown next to the branches (Felsenstein, 1985). Initial tree(s) for the heuristic search were obtained automatically by applying Neighbor-Join and BioNJ algorithms to a matrix of pairwise distances estimated using the JTT model, and then selecting the topology with superior log likelihood value. A discrete Gamma distribution was used to model evolutionary rate differences among sites (5 categories (+G, parameter = 0.6168)). This analysis involved 13 amino acid sequences. All positions with less than 95% site coverage were eliminated, i.e., fewer than 5% alignment gaps, missing data, and ambiguous bases were allowed at any position (partial deletion option). There were a total of 11690 positions in the final dataset. A species tree generated in Orthofinder was used to confirm the topology of the ML tree. The species tree was inferred from a set of unrooted orthogroup gene trees using STAG (Emms & Kelly, 2018) and rooted using STRIDE Emms & Kelly 2017). The ML tree was then converted into an ultrametric tree and calibrated based on a divergence time of 105 Ma between *A. thaliana* and *P. trichocarpa* (Clarke *et al.* 2011) in MEGA X (Kumar *et al.,* 2018) using the RelTime method (Tamura *et al.* 2012). The ultrametric tree and the gene numbers in each orthogroup from the OrthoFinder results were then used for the CAFE analysis. The functional annotation of each OG was predicted based on sequence similarity to the InterPro protein family database where the majority of the proteins in the OG shared the same Pfam annotation.

Prediction, analysis and refinement of the *S. hermonthica* secretome

Secreted *S. hermonthica* proteins were predicted according to the default thresholds using SignalP3.0 and SignalP4.1 (Bendtsen *et al.,* 2004; Petersen *et al.,* 2011) (Fig. S1). SignalP3.0 predicts a secretion signal peptide according to both neural network (SignalP3.0-NN) and hidden Markov model (SignalP3.0-HMM) algorithms, whilst SignalP4.1 uses a neural network algorithm. Putative secreted proteins were searched for transmembrane spanning regions in the remaining portion of the protein sequence (without secretion signal) using TMHMM2.0 (Krogh *et al.* 2001). Proteins with a secretion signal but without a predicted transmembrane helix were retained as the 'secretome'. Putative secreted proteins were ranked according to the agreement between the different SignalP algorithms: '3' = predicted by all three SignalP algorithms (SignalP3.0-NN, SignalP3.0-HMM and SignalP4.1); '2' = predicted by at least two algorithms and '1' = predicted by one algorithm. TargetP1.1 was used to provide additional predictions on the cellular localization of the putative secreted proteins ('S' = extracellular; 'C' = chloroplast; 'M' = mitochondrial) (Emanuelsson *et al.,* 2000). The closest matching *Arabidopsis thaliana* protein for

each *Striga hermonthica* putative secreted protein was queried against the SUBA4 online database, which combines bioinformatic and experimental evidence to predict subcellular localisation of *A. thaliana* proteins (https://suba.live) (Hooper *et al.,* 2017). The consensus location of each queried protein was recorded and the number of each subcellular location was expressed as a percentage of the total. Using this approach, the most common subcellular location predicted in the secretome was 'extracellular', supporting the validity of our secretome prediction pipeline for *S. hermonthica* (Table S5).

To determine whether a putative secreted protein was expressed during early stages of parasitism a BLASTn search using the protein's coding sequence was conducted against the stage 3 (~ 48 hours post infection) and stage 4 (~ 72 hours post infection) transcriptome dataset downloaded from the PPGP (Lu *et al.,* 2016) BLASTn hits were taken to be significant if they returned an e-value < 10^{-10}, percentage identity of > 95 % and a bit score of > 60. To identify enriched Pfam domains in the *S. hermonthica* secretome compared with the rest of the proteome (non-secretome), the frequency of each Pfam domain was first calculated as the number of occurrences of each domain over the total number of domains in the secretome or non-secretome. Enrichment fold for each domain was then calculated as the frequency of hits in the secretome over the frequency in the non-secretome. The significance of enrichment was assessed using a Chi-squared test with a false discovery rate (FDR) correction for multiple testing (Benjamini *et al.,* 1995) and was taken to be significant when the corrected p value was < 0.1.

The initial secretome was refined into subsets based on a series of structural and functional characteristics (Fig. S1). First, those putative secreted proteins that met the following criteria were retained: SignalP ranking = 3; TargetP location = S; and have a BLASTn hit against either stage 3 or 4 *S. hermonthica* transcriptome libraries (suggesting gene expression during early stages of parasitism). From these, four subsets of proteins were identified. Subset 1 comprised small, secreted cysteine-rich proteins (SSCRPs) $<$ 500 amino acids in length and containing $\geq$ 4 % cysteine residues. Subset 2 consisted of proteins containing Pfam domains that were significantly enriched in the *S. hermonthica* secretome. Only those Pfam domains that were enriched to a greater extent in the *S. hermonthica* secretome compared with their enrichment in the *M. guttatus* secretome (determined in exactly the same way as for *S. hermonthica*) were used to obtain this subset. Subset 3 comprised proteins with no known Pfam domains or orthogroup assignment. Subset 4 contained proteins with similarity to proteins involved in host-pathogen interactions based on having a match against the PHI-base (Fig. S1).

To test whether genes in the secretome clustered together in the genome, a permutation test was performed. For each gene, 'MinDis', defined as the distance between the start positions of the focal gene and its nearest neighbouring gene, was calculated. Genes without a neighbouring gene on the same scaffold were excluded. MinDis was also calculated for 10,000 randomly sampled gene sets with the same size as the secretome. The numbers of clustered genes with MinDis falling into a series of distance intervals were counted and were then compared with the numbers in the same distance interval in the 10,000 random gene sets. (JAVA scripts are provided in Note S1.

Identification of and analysis of candidate virulence loci using pooled sequencing data

Trimming and filtering: The raw sequence reads from the six pools were trimmed to remove low quality bases and adapter sequences using CutAdapt (Martin 2011). The cleaned reads were mapped to the *S. hermonthica* reference genome using both BWA mem v 0.7.15 (Li & Durbin 2010) and NOVOALIGN (http://www.novocraft.com). Mapped reads were sorted using Samtools (Li *et al.,* 2009) and PCR duplicates were removed using Picard (http:// broadinstitute .github .io /picard/). Reads around indels were realigned using the modules RealignerTargetCreator and IndelRealigner implemented in gatk (Van der Auwera *et al.,* 2013). SNPs were called using bcftools (https://samtools. github.io/bcftools/). An R script described in (Morales *et al.,* 2019) was used to infer the read coverage distribution for each SNP for each pool of sequenced reads, based on three-component mixture models. An upper boundary of the coverage for the intermediate component (single and low copy number regions) was obtained from the fitted distribution for each pool. The high-coverage component is expected to be enriched in repetitive sequences while the low coverage component is enriched in erroneous reads or inadequately sampled genome regions. In order to provide robust data for subsequent analyses, the inference of an allele and its frequency was considered to be reliable only if the following criteria were met. (a) Coverage from each pool was ≥ 4 and $\leq$ the upper boundary inferred from the mixture model distribution. (b) The sum of the coverage across the six DNA pools was ≥ 36. (c) The count of the minor allele frequency, summed over pools, was > 2. (d) The ratio of the coverage of the second highest allele to the total coverage was ≥ 0.1. (e) The ratio of the combined coverage of the third and fourth highest alleles to the total coverage was < 0.02. Only the highest and second highest frequency alleles were considered for the subsequent analyses. Although three or four alleles were present at some positions, the additional alleles were always rare. Cut-offs were determined following exploration of a range of alternative values. They removed only variants that are very unlikely to differ significantly among pools and so they did not influence the ranking of candidate loci.

Linkage disequilibrium: Firstly, the distribution of the gene lengths, i.e. from the start codon to the stop codon of a gene, for all the genes in the *S. hermonthica* genome was obtained and 4 kbp, which is the 75th percentile of the gene length distribution, was used as the upper limit for the length of region to be considered. The SNP with maximum ΔAICcv within a gene was identified and the region up to 4 kbp from the SNP was divided into eight 500 bp intervals. The mean difference of ΔAICcv, relative to the maximum ΔAICcv, for SNPs in each 500 bp interval was then calculated. The mean difference was typically only elevated in the 500 bp interval containing the SNP with maximum ΔAICcv, so it was clear that linkage disequilibrium above background levels did not normally extend beyond 1 kbp (see Fig. S2). Therefore, 1 kbp windows were used to detect genomic regions.

Detection of candidate loci: Regions starting from 5kbp upstream of the start codon and ending no further than 2 kbp downstream of the stop codon of a gene were divided into 1 kbp-windows and the mean ΔAICcv across all the SNPs in each window was calculated. A permutation test was performed to obtain the probability of observing the mean ΔAICcv value, or higher, for each window based on the distribution of ΔAICcv across the regions as a whole.

A mean ΔAICcv was calculated for a window containing n SNPs. For the permutation test, one hundred thousand samples of n SNPs from the pool of all SNPs within the analysed regions genome-wide, were drawn randomly. For each of these samples, a mean ΔAICcv value was calculated and an expected distribution for the mean in the target window was obtained. The probability that the observed mean ΔAICcv of a window could be matched or exceeded by chance was obtained by counting the number of permuted mean ΔAICcv with values lower than the observed mean ΔAICcv value for that window and then divided by 100,000. The R and JAVA scripts are provided in Notes S2 and S3.

Finally, we retained genic regions (defined as regions from 2 kbp upstream of the start codon to the 1 kbp window containing the stop codon) for which this probability was less than or equal to $2x10^{-5}$ for both the BWA and NOVOALIGN analyses in any window. This cut-off was chosen to provide experiment-wide significance given the number of protein-coding genes in the analysis (29,518). In the secretome, a more relaxed cut-off of $1x10^{-4}$ was used to reflect the prior expectation that the secretome would be enriched with pathogenicity-related genes and the smaller number of genes in this set (3,375). Thirty-two genes met this criterion for both Novoalign and BWA (Data S1). In addition, six genes encoding putative secreted proteins that passed the $1x10^{-4}$ cut-off for either Novoalign or BWA were included in the candidate set because they either contained large numbers of non-synonymous SNPs or contained high impact SNPs that can alter protein structure (e.g. due to protein truncation) (Data S1).

Population statistics: Two population statistics, nucleotide diversity (π) and Tajima's D, were calculated for each genic region in the control pool, using the perl script 'Variance-at-position.pl' implemented in the software Popoolation (Kofler *et al.,* 2011). The mpileup file combining the three control pools was generated in Samtools (Li *et al.,* 2009). Minimum counts for minor alleles were set to two to account for sequencing errors. The minimum coverage per site was set to 12 and the maximum coverage was set to 220, which was inferred from the mixture model distribution. The pool size was set to 600 because 300 diploid genomes were represented in the combined control pool.

Divergence measure: To provide a measure of divergence per gene, we used the proportion of SNPs with high F_{ST}. The F_{ST} values between the control and virulent pools for each SNP were calculated using Popoolation2 (Kofler *et al.,* 2011). The 95th percentile was obtained from the F_{ST} values for all SNPs across the genic regions as a whole. For each genic region, we counted the number of SNPs in its significant window(s) with F_{ST} value higher than the 95th percentile and then divided by the total number of SNPs in the significant window. This provided a measure of differentiation for each candidate virulence gene between the *S. hermonthica* grown on the two host varieties.

Expression profiling of candidate virulence genes

To determine the expression profiles for candidate virulence genes, an RNA-seq analysis was conducted for *S. hermonthica* (Kibos accession) collected at 2, 4, or 7 days post infection on NERICA 7. Rice plants were grown and infected in the rhizotron system as described previously (Gurney et al., 2006). S. *hermonthica* were collected from the roots of NERICA 7 by cutting a 1 mm section of rice root around each *S. hermonthica* attachment. In addition, unattached *S. hermonthica* haustoria were induced *in vitro* by the addition of 10 µM DMBQ (Fernández-Aparicio

et al., 2013). For each treatment, four biological replicates were generated. RNA was extracted using the RNeasy plant Minikit (Qiagen) and 2 µg of RNA was treated with DNase to remove DNA contamination (dsDNase, Thermoscientific). The resulting RNA was purified and concentrated using the RNeasy MinElute cleanup kit (Qiagen) and then sent to NovoGene Co. Ltd. (China) for sequencing (https://en.novogene.com/). Cleaned reads were mapped to the *S. hermonthica* genome using Tophat2, version v2.0.12 (default settings except 'mismatch' = 2). Transcript abundance was determined as read count and measured by HTSeq (version 0.6.1) with default settings, except that the '-m' option was set to 'union'. The FPKM values for each gene at each time point were used to calculate a fold change in expression relative to the haustorial sample. To determine whether gene expression changed significantly during infection, a one-way ANOVA was carried out for each gene using the aov function in R (R Core Team, 2021). If the assumptions of ANOVA were not met, data were log transformed. For each gene, log2 fold expression values, across the time points, were centred around 0 and scaled by the standard deviation for plotting as a heatmap. Heatmap plotting was conducted using the pheatmap function in R (R Core Team, 2021) and clustering of genes by expression profiles was conducted using the 'complete' method with the 'Euclidean' distance measure.

References

Bendtsen J D, Nielsen H, von Heijne G, Brunak S. **2004**. Improved prediction of signal peptides: SignalP 3.0. *Journal of Molecular Biology* **340**: 783–795.

Benjamini Y, Hochberg Y. **1995**. Controlling the false discovery rate: A practical and powerful approach to multiple testing. *Journal of the Royal Statistical Society: Series B (Methodological)* **57**: 289–300.

Birney E. **2004**. GeneWise and genomewise. *Genome Research* **14**: 988–995.

Clarke JT, Warnock RCM, Donoghue PCJ. **2011**. Establishing a time-scale for plant evolution. *New Phytologist* **192**: 266–301.

Emanuelsson O, Nielsen H, Brunak S, von Heijne G. **2000**. Predicting subcellular localization of proteins based on their N-terminal amino acid sequence. *Journal of Molecular Biology* **300**: 1005–1016.

Emms DM, Kelly S. **2015**. OrthoFinder: solving fundamental biases in whole genome comparisons dramatically improves orthogroup inference accuracy. *Genome Biology* **16:** 157 doi:10.1186/s13059-015-0721-2

Emms DM, Kelly S. 2017. STRIDE: species tree root inference from gene duplication events. *Molecular Biology and Evolution* **34:** 3267–78.

Emms DM, Kelly S. 2018. STAG: species tree inference from all genes. bioRxiv. doi: https://doi.org/10.1101/267914

Felsenstein J, 1985. Confidence limits on phylogenies: An approach using the bootstrap. *Evolution* **39:** 783-791.

Fernández-Aparicio M, Huang K, Wafula EK, Honaas LA, Wickett NJ, Timko MP, dePamphilis CW, Yoder JI, Westwood JH. **2013**. Application of qRT-PCR and RNA-Seq analysis for the

identification of housekeeping genes useful for normalization of gene expression values during Striga hermonthica development. *Molecular Biology Reports* **40**: 3395–3407.

Gurney AL, Slate J, Press MC, Scholes JD. **2006**. A novel form of resistance in rice to the angiosperm parasite *Striga hermonthica*. *New Phytologist* **169**: 199–208.

Han MV, Thomas GWC, Lugo-Martinez J, Hahn MW. **2013**. Estimating gene gain and loss rates in the presence of error in genome assembly and annotation using CAFE 3. *Molecular Biology and Evolution* **30**: 1987–1997.

Haas BJ, Salzberg SL, Zhu W, Pertea M, Allen JE, Orvis J, White O, Buell CR, Wortman JR. **2008**. Automated eukaryotic gene structure annotation using EVidenceModeler and the Program to Assemble Spliced Alignments. *Genome Biology* **9**: R7.

Heo Y, Wu X-L, Chen D, Ma J, Hwu W-M. **2014**. BLESS: Bloom filter-based error correction solution for high-throughput sequencing reads. *Bioinformatics* **30**: 1354–1362.

Hoff KJ, Lange S, Lomsadze A, Borodovsky M, Stanke M. **2016**. BRAKER1: Unsupervised RNA-Seq-based genome annotation with GeneMark-ET and AUGUSTUS: *Bioinformatics* **32**: 767–769.

Hooper CM, Castleden IR, Tanz SK, Aryamanesh N, Millar AH. **2017**. SUBA4: the interactive data analysis centre for Arabidopsis subcellular protein locations. *Nucleic Acids Research* **45**: D1064–D1074.

Jones DT, Taylor WR, Thornton JM. 1992. The rapid generation of mutation data matrices from protein sequences. *Computer Applications in the Biosciences* **8:** 275-282.

Katoh K, Standley DM. **2013**. MAFFT Multiple Sequence Alignment Software Version 7: Improvements in performance and usability. *Molecular Biology and Evolution* **30**: 772–780.

Kofler R, Orozco-terWengel P, De Maio N, Pandey RV, Nolte V, Futschik A, Kosiol C, Schlötterer C. **2011**. PoPoolation: A toolbox for population genetic analysis of next generation sequencing data from pooled individuals. *PLoS ONE* **6**: e15925.

Krogh A, Larsson B, von Heijne G, Sonnhammer ELL. **2001**. Predicting transmembrane protein topology with a hidden markov model: application to complete genomes. *Journal of Molecular Biology* **305**: 567–580.

Kumar S, Stecher G, Li M, Knyaz C, Tamura K. **2018**. MEGA X: Molecular Evolutionary Genetics Analysis across computing platforms. *Molecular Biology and Evolution* **35**: 1547–1549.

Li H, Durbin R. **2010**. Fast and accurate long-read alignment with Burrows–Wheeler transform. *Bioinformatics* **26**: 589–595.

Li H, Handsaker B, Wysoker A, Fennell T, Ruan J, Homer N, Marth G, Abecasis G, Durbin R, 1000 Genome Project Data Processing Subgroup. **2009**. The Sequence Alignment/Map format and SAMtools. *Bioinformatics* **25**: 2078–2079.

Lu S, Edwards MC. **2016**. Genome-Wide Analysis of Small Secreted Cysteine-Rich Proteins Identifies Candidate Effector Proteins Potentially Involved in *Fusarium graminearum* −Wheat Interactions. *Phytopathology* **106**: 166–176.

Martin M. 2011. Cutadapt removes adapter sequences from high-throughput sequencing reads. *EMBnet.journal* **17:** 10-12.

Morales HE, Faria R, Johannesson K, Larrson T, Panova M, Westram A M, Butlin R K. **2019**. Genomic architecture of parallel ecological divergence: Beyond a single environmental contrast. *Science Advances*: eaav9963.

Petersen TN, Brunak S, von Heijne G, Nielsen H. **2011**. SignalP 4.0: discriminating signal peptides from transmembrane regions. *Nature Methods* **8**: 785.

R Core Team (2021). R: A language and environment for statistical computing, Vienna, Austria. Version 4.0.2 URL http://www.R-project.org/

Stewart CN, Via LE. 1993. A rapid CTAB DNA isolation technique useful for RAPD fingerprinting and other PCR applications. *Biotechniques* 14: 748–750.

Tamura K, Battistuzzi FU, Billing-Ross P, Murillo O, Filipski A, and Kumar S. 2012. Estimating Divergence Times in Large Molecular Phylogenies. *Proceedings of the National Academy of Sciences* **109**: 19333-19338.

Trapnell C, Pachter L, Salzberg SL. **2009**. TopHat: discovering splice junctions with RNA-Seq. *Bioinformatics* **25**: 1105–1111.

Trapnell C, Roberts A, Goff L, Pertea G, Kim D, Kelley DR, Pimentel H, Salzberg SL, Rinn JL, Pachter L. **2012**. Differential gene and transcript expression analysis of RNA-seq experiments with TopHat and Cufflinks. *Nature Protocols* **7**: 562–578.

Van der Auwera GA, Carneiro MO, Hartl C, Poplin R, del Angel G, Levy-Moonshine A, Jordan T, Shakir K, Roazen D, Thibault J, *et al.* **2013**. From FastQ data to high-confidence variant calls: The Genome Analysis Toolkit best practices pipeline. In: Bateman A, Pearson WR, Stein LD, Stormo GD, Yates JR, eds. *Current Protocols in Bioinformatics* **43**: 11.10.1-11.10.33 Hoboken, NJ, USA: John Wiley & Sons, Inc.

Westwood JH, dePamphilis CW, Das M, Fernández-Aparicio M, Honaas LA, Timko MP, Wafula EK, Wickett NJ, Yoder JI. **2012**. The parasitic plant genome project: New tools for understanding the biology of Orobanche and Striga. *Weed Science* **60**: 295–306.

Xu H, Luo X, Qian J, Pang X, Song J, Qian G, Chen J, Chen S. **2012**. FastUniq: A Fast De Novo Duplicates Removal Tool for Paired Short Reads. *PLoS ONE* **7**: e52249.

Yang Z, Wafula EK, Honaas LA, Zhang H, Das M, Fernandez-Aparicio M, Huang K, Bandaranayake PCG, Wu B, Der JP, *et al.* **2015**. Comparative transcriptome analyses reveal core parasitism genes and suggest gene duplication and repurposing as sources of structural novelty. *Molecular Biology and Evolution* **32**: 767–790.
